# Autophagy-modulating biomaterials: multifunctional weapons to promote tissue regeneration

**DOI:** 10.1186/s12964-023-01346-3

**Published:** 2024-02-15

**Authors:** Yan Wu, Luxin Li, Zuojun Ning, Changrong Li, Yongkui Yin, Kaiyuan Chen, Lu Li, Fei Xu, Jie Gao

**Affiliations:** 1 Heilongjiang Key Laboratory of Tissue Damage and Repair, Mudanjiang Medical University, Mudanjiang, 157000 China; 2 Changhai Clinical Research Unit, Shanghai Changhai Hospital, Naval Medical University, Shanghai, 200433 China; 3 Department of plastic surgery, Naval Specialty Medical Center of PLA, Shanghai, 200052 China

**Keywords:** Anti-apoptosis, Anti-infection, Anti-inflammation, Autophagy, Biomaterials, Proliferation and differentiation, Tissue regeneration

## Abstract

Autophagy is a self-renewal mechanism that maintains homeostasis and can promote tissue regeneration by regulating inflammation, reducing oxidative stress and promoting cell differentiation. The interaction between biomaterials and tissue cells significantly affects biomaterial-tissue integration and tissue regeneration. In recent years, it has been found that biomaterials can affect various processes related to tissue regeneration by regulating autophagy. The utilization of biomaterials in a controlled environment has become a prominent approach for enhancing the tissue regeneration capabilities. This involves the regulation of autophagy in diverse cell types implicated in tissue regeneration, encompassing the modulation of inflammatory responses, oxidative stress, cell differentiation, proliferation, migration, apoptosis, and extracellular matrix formation. In addition, biomaterials possess the potential to serve as carriers for drug delivery, enabling the regulation of autophagy by either activating or inhibiting its processes. This review summarizes the relationship between autophagy and tissue regeneration and discusses the role of biomaterial-based autophagy in tissue regeneration. In addition, recent advanced technologies used to design autophagy-modulating biomaterials are summarized, and rational design of biomaterials for providing controlled autophagy regulation via modification of the chemistry and surface of biomaterials and incorporation of cells and molecules is discussed. A better understanding of biomaterial-based autophagy and tissue regeneration, as well as the underlying molecular mechanisms, may lead to new possibilities for promoting tissue regeneration.

Video Abstract

**Supplementary Information:**

The online version contains supplementary material available at 10.1186/s12964-023-01346-3.

## Introduction

Autophagy is an evolutionarily conserved mechanism for the degradation of cytosolic proteins and organelles through lysosomal degradation. Autophagy is classified as macroautophagy, microautophagy, and chaperone-mediated autophagy (CMA) [1], all of which involve the proteolytic degradation of cytosolic components in lysosomes. The process of macroautophagy involves the transfer of cytoplasmic cargo to lysosomes by means of an autophagosome, a double membrane-bound vesicle. Conversely, microautophagy occurs when a lysosome invaginates the membrane, thereby directly absorbing cytosolic components. In CMA, targeted proteins are translocated across the lysosome membrane by chaperone proteins that can be recognized by the lysosomal membrane receptor lysosomal-associated membrane protein 2 A, causing the unfolding and degradation of the complex. This review focuses on macroautophagy (henceforth referred to as ‘autophagy’). Autophagy at low levels occurs in all cells and is considered a mechanism for maintaining normal cellular homeostasis. As a protective mechanism, autophagy is upregulated in response to several types of stress stimuli, including starvation, oxidative stress, hypoxia, and infection. This adaptive response protects the cell membrane and organelles from genomic damage and metabolic stress, prevents tumor development, and maintains cellular homeostasis by recycling nutrients and energy under unfavorable conditions [2]. Autophagy is regulated by a set of genes collectively called autophagy-related genes (ATGs), such as LC3 and Beclin-1, which have been identified in yeast [3]. These genes perform various functions within the cell, including regulation of intracellular communication and nonautophagic pathways [4]. Disruption of autophagy inhibits ubiquitination, promotes the accumulation of reactive oxygen species (ROS), reduces mitochondrial function, and increases genomic instability, leading to a decrease in the quality of intracellular components [5]. Therefore, dysregulation of autophagy is involved in the pathogenesis of various conditions and diseases, including tissue damage.

Tissue regeneration, which is a complex and metabolically demanding process, involves restoring the function and structure of injured tissues. The ability to regenerate tissues effectively is essential for the survival of all living organisms [6]. An increase in the elderly population and the incidence of accidents and other trauma injuries have expanded the need for tissue regeneration. During the 2018–2023 period, the global tissue engineering and regeneration market grew from $24.7 billion to $109.9 billion at a compound annual growth rate (CAGR) of 34.8% (https://www.medgadget.com/). Additionally, the coordinated process of tissue regeneration requires precise spatiotemporal mechanisms of action [7]. However, based on the regenerative ability of tissues, the outcome of tissue regeneration is usually imperfect and is often accompanied by fibrosis resulting from abnormal accumulation.

Autophagy is involved in the complex process of tissue regeneration, including the interactions between functional cells and stromal and immune cells. For example, the activation of autophagy and myofibroblast differentiation during tissue regeneration are positively correlated [8]. Furthermore, through autophagy, recombinant retinyl esters stored in lipid droplets are cleaved to generate fatty acids, which stimulate the myofibroblast differentiation of hepatic stellate cells (HSCs). It has also been observed that autophagy promotes cellular homeostasis in liver endothelial cells, macrophages, and hepatocytes [8]. Furthermore, it plays a crucial role in maintaining long-lived cells, such as neurons, cardiomyocytes, and osteocytes [9]. After proteins enter cells through endocytosis, they can be stored in the trans-Golgi network by retromers, such as Beclin-1, or in late endosomes. Subsequently, they are degraded by hydrolytic enzymes after their merging with lysosomes [10]. The accumulation of toxic and lethal aggregates following this process can damage molecules and lead to cell death if these charged aggregates are not degraded properly due to pH fluctuations or other physiological imbalances. It has also been observed that autophagy dysfunction can trigger various pathophysiological conditions, including brain disorders, diabetes, cardiovascular disease, viral infection, and cancer [11]. Additionally, autophagy plays a crucial role in infection and inflammation by removing intracellular bacteria, promoting the production of inflammatory cytokines, controlling inflammation, and driving antigen presentation [12, 13]. When a tissue is injured, necrotic debris as well as clotting and invading microbes trigger inflammatory responses, which can then be propagated by the chemotactic factors released locally by inflammatory cells. Neutrophils, monocytes, and other innate immune cells are also recruited to the wound site to remove debris and kill pathogens during tissue regeneration. It is also worth noting that a key role of autophagy in tissue regeneration is the modulation of innate and adaptive immune responses in macrophages [14]. Proinflammatory macrophages adopt a reparative phenotype during this period, resulting in reduced inflammation. Eventually, tissue homeostasis is restored. The degree and duration of the response differ at each stage, which influences the outcome of tissue regeneration [15]. Reportedly, many pathogens have evolved ways by which they evade the autophagic machinery by releasing factors that interfere with the maturation of autophagosomes, thereby preventing the fusion of autophagosomes with lysosomes and competing with the autophagy receptors of the host for binding to LC3 [16]. Therefore, autophagy plays a critical role in tissue regeneration, and a strategy for its modulation represents a promising approach to promoting tissue regeneration.

Biomaterials with excellent biocompatibility and multiple modification sites have been used for tissue regeneration as versatile therapeutic agents [17, 18]. Nanomaterials (NMs) are increasingly essential in the field of regenerative medicine and the advancement of therapeutic approaches for diverse diseases [19]. The presence of a significant level of reactive oxygen species (ROS) frequently hampers the viability, maintenance, and differentiation potential of stem cells. Cerium oxide nanoparticles (CeNMs) exhibit oxygen-modulating characteristics. The findings suggest that the utilization of CeNMs holds promise in safeguarding stem cells and endothelial cells against the detrimental effects of ROS-rich environments [20, 21]. Creating a conducive environment for osteogenesis is a crucial approach in the development of stem cell-derived bone-equivalent tissues. The FGF18-BGn/Col gel is widely regarded as a highly effective osteopromoting reservoir that facilitates the support and signaling of MSCs in the field of bone tissue engineering [22]. In order to effectively engineer interfacial tissue between bone and cartilage, it is necessary to address the distinct properties and structures of the osteochondral region. Two types of three-dimensional scaffolds, characterized by different surface topographies, namely “dense” and “nanofibrous,“ have been found to elicit varying responses from osteo- and chondrocytes. The dense scaffold is primarily utilized for the chondral component, while the nanofibrous structure is more suitable for the osteo component, thereby creating an ideal biphasic matrix environment for osteochondral tissue engineering [23]. The interaction between biomaterials and tissue cells significantly affects biomaterial-tissue integration and tissue regeneration [24]. Because mammalian cells possess a complex quality-control system that prevents the accumulation of aberrant materials, such as proteinaceous aggregates, they can clear such materials by activating degradation pathways [25]. Biomaterials entering cells are likely to be considered foreign bodies, which may activate clearance mechanisms, such as autophagy [26]. Several mechanisms are associated with the use of biomaterials to regulate autophagy. Due to their different physicochemical properties, the underlying molecular mechanisms by which biomaterials activate autophagy are diverse, including direct modulation of important signaling molecules, such as AMPK, mTOR, and PI3K/Akt [27, 28]. Studies have also shown that biomaterials of different sizes and compositions are particularly effective in modulating autophagy, which is the main catabolic process responsible for degrading bulk intracellular materials [29]. Therefore, it is necessary to investigate the autophagy-altering functions of biomaterials, especially their benefits and safety. Biomaterials can also affect the various processes that are related to tissue regeneration by regulating autophagy. The effects of these biomaterials on inflammatory response regulation, oxidative stress, cell differentiation, proliferation, migration, apoptosis, and the formation of the extracellular matrix may be key factors that determine how biomaterials improve tissue regeneration. In addition, biomaterials may regulate autophagy in a time- and dose-dependent manner. For example, Li et al. observed that Al_2_O_3_ particles induce the autophagy of fibroblasts in a dose- and time-dependent manner in vitro and in vivo. In contrast, another study showed that decreasing autophagy levels induce osteolysis and aseptic prosthetic loosening, while increasing autophagy levels reverted these effects [30]. Taken together, biomaterial-based autophagy regulation in a time- and space-dependent manner could be expected to facilitate tissue regeneration. Furthermore, recent studies have also demonstrated the efficacy of biomaterials in augmenting drug delivery mechanisms and optimizing therapeutic outcomes [31, 32]. These materials are employed to enhance the efficiency of drug transportation, bolster targeted attributes, and mitigate potential adverse effects [33]. Xu et al. conducted a study wherein they formulated and produced PLGA microspheres with the purpose of delivering platelet-derived growth factor (PDGF) to expedite the process of wound healing by suppressing autophagy [34]. Huang et al. devised and fabricated a DNA nanostructure-mediated nanoplatform, incorporating spermidine and siRNA, with the aim of treating acute lung injury (ALI). The utilization of the endogenous polyamine, spermidine, in conjunction with mTOR siRNA, was found to augment macrophage autophagy [17]. Collectively, Biomaterials possess the capability to facilitate tissue damage repair via autophagy, while also demonstrating their efficacy through autophagic drug administration.

Over the past decade, autophagy-modulating biomaterials have attracted considerable attention owing to their unique advantages in tissue regeneration, and several preclinical studies have reported encouraging results. In addition, many reviews have reported the potential and significance of autophagy in tissue regeneration [25, 35–38]. However, to the best of our knowledge, no review has provided a comprehensive summary of autophagy-modulating biomaterials. In this review, we summarized the recent progress on the use of autophagy-modulating biomaterials in tissue regeneration based on a literature review. In addition, we discuss the relationship between autophagy and tissue regeneration and the role of various types of biomaterials in promoting the regeneration of organs by regulating autophagy. Understanding the relationship between tissue regeneration and autophagy-modulating biomaterials and the underlying molecular mechanisms may offer new potential strategies for promoting tissue regeneration.

## The process and molecular mechanisms of autophagy

### The process of autophagy

Autophagy is a complex and dynamic multistep process regulated by ATGs. Nutrients, energy, and stress-sensing mechanisms within the cell affect autophagy by activating and inhibiting ATGs [39]. After ATG complexes are activated, cytoplasmic cargoes are captured in double-membrane vesicles called autophagosomes. These cargoes contain damaged or excessive proteins, organelles, lipids, and glycogen, which are tagged with ubiquitin and recognized by autophagy receptors called sequestasome 1 (p62). LC3II is a component of the autophagosome membrane that facilitates sequestration of cargoes through cargo receptors. After the fusion of autophagosomes and lysosomes into the autophagosome-lysosome complex, the cargoes are degraded by hydrolases. As a result of degradation, amino acids, nucleotides, and free fatty acids are introduced into the energy cycle and can be reused to maintain the normal metabolism of cells. Therefore, autophagy is essential for maintaining cellular metabolism, energy homeostasis, and survival during starvation and preventing the accumulation of toxins in damaged proteins and organelles [40].

### Autophagy-related genes and proteins

Autophagy-related genes and proteins are considered the core regulators of autophagosome biogenesis [41]. They have been identified in multiple organisms, from yeasts to mammals, and have similar mechanisms. During autophagosome nucleation, class III phosphatidylinositol 3 kinase (PI3K) interacts with Beclin-1 to form the Beclin-1/PI3K complex, which plays a role in targeting ATGs to phagocytes. Elongation of the phagophore membrane is regulated by two ubiquitin-like complexes, namely, ATG12-ATG5-ATG16L1 and microtubule-associated protein 1 light chain 3 (MAP1LC3)/LC3. Under the action of ATG3 and ATG7, LC3I and phosphatidylethanolamine form LC3II before autophagy is induced. LC3II strongly binds to the membranes of phagophores and autophagosomes and serves as a marker for autophagosome formation; therefore, the LC3II protein is widely used to monitor autophagy.

Noncanonical autophagy pathways lead to the degradation of autophagosomes by evading the canonical pathway [42]. Various noncanonical autophagy pathways have been discovered, such as Beclin-1-independent autophagy, which does not depend on proteins involved in phagophore nucleation. Similarly, some noncanonical pathways do not require proteins involved in phagophore extension and closure (such as ATG5, ATG7, and LC3). Some studies have described LC3-associated phagocytosis (LAP) as a noncanonical autophagy pathway. Unlike canonical autophagy, LAP involves the conjugation of LC3 to phosphatidylethanolamine on the single-membrane phagosome through some components of the autophagy machinery (such as ATG5, ATG7, ATG12, and ATG16L1 for LC3 lipidation). Lipidated LC3II eventually facilitates lysosomal fusion. LAP increases immunity and helps phagocytic cells, including macrophages, clear extracellular particles and pathogens [41].

To date, more than 40 ATGs encoded by yeast have been identified [43]. Autophagy in mammalian cells is regulated by approximately 20 core ATGs, which are divided into different functional units as follows: the core ULK complex (ULK1/2, ATG13, RB1CC1/FIP200, and ATG101), the autophagy-specific PI3K complex (VPS34, VPS15, Beclin-1, and ATG14 L), the ATG9A trafficking system (ATG9A, WIPI1/2, and ATG2A), the ATG12 ubiquitin-like conjugation system (ATG12, ATG7, ATG10, ATG5, and ATG16L1), and the LC3 ubiquitin-like conjugation system (LC3A/B/C, ATG7, ATG3, and ATG4A/B/C/D). ATGs are recruited hierarchically to the vacuole and form preautophagosomal structures (PASs), which are necessary for the formation of autophagosomes [44].

### Role of autophagy in tissue regeneration

Regeneration of tissues requires autophagy to regulate tissue remodeling; however, the effects of autophagy can vary depending on the type of tissue and duration of the initial injury [37]. Diverse cell events can induce autophagy, such as nutrient deprivation, hypoxia, oxidative stress, inflammation, and infection [41], and cell injury can be caused by any of these factors. Activated autophagy promotes cellular health and survival during tissue regeneration by exerting antioxidation, anti-inflammation, anti-infection, and anti-apoptosis effects while promoting cell proliferation and differentiation. However, excessive stimulation of autophagy can cause cell damage [45]. If lysosomal clearance of autophagy fails, the activation of autophagy will lead to cell traffic jams, resulting in an increase in the pathology of aging, Parkinson’s disease, and other neuro- and myodegenerative disorders [46]. Therefore, autophagy at moderate levels regulates and stabilizes the intracellular environment, whereas excessive autophagy may lead to cell death and tissue damage [14]. The effects of autophagy on tissue regeneration differ based on tissues, cells, and stimuli.

### Autophagy activation eliminates ROS

The presence of small amounts of ROS has beneficial biological effects, including angiogenesis, wound healing, elimination of pathogenic organisms, and tissue regeneration [47]. However, if ROS production is excessive, ROS-mediated oxidative stress can cause damage to biological macromolecules (such as proteins, lipids, and nucleic acids). Oxidized macromolecules can disrupt cellular homeostasis and functions, thus causing damage to many organs [48]. ROS can trigger autophagy early during oxidative stress, contributing to antioxidant defenses [49]. For example, stimulating factors such as starvation, pathogens, or death receptors can activate ROS and trigger autophagy, which eliminates damaged mitochondria through lysosome-based degradation [50]. Negative feedback mechanisms involving autophagy and ROS help to mitigate oxidative stress and promote cell survival [50]. However, impaired autophagy leads to ROS accumulation, resulting in oxidative stress [51]. Because antioxidant molecules can partially or completely inhibit autophagy [52], mitochondrial ROS can simultaneously stimulate and inhibit autophagic signaling. Although ROS and autophagy are mutually influenced, monotherapy with either antioxidants or autophagy activators is unsuccessful in treating diseases associated with dysfunctional autophagy and oxidative stress. Several studies have investigated combination therapies that regulate both antioxidant pathways and autophagy [53].

### Autophagy regulates the anti-inflammatory response

Inflammatory responses protect tissues against pathogens, toxins, and tissue damage, both endogenous and exogenous [54]. In any cell that is capable of activating a cell-autonomous inflammatory response, the cytoplasmic cleaning function of autophagy is anti-inflammatory by default. Autophagy can also reduce inflammasome activation and NF-κB by removing damaged mitochondria, thus preventing excessive inflammatory reactions and tissue damage [55]. During inflammatory conditions, macrophages play a crucial role in the initiation, maintenance, and resolution of inflammation. Macrophages are heterogeneous cells that can differentiate into different phenotypes depending on their microenvironment. M1 macrophages kill pathogens and release inflammatory factors such as IL-1β, IL‐6, and TNF‐α during the early stage of inflammation, promote ROS production and finally cause apoptosis [56], whereas M2 macrophages suppress immune responses and promote tissue regeneration during the late stage of inflammation [57, 58]. Autophagy is an essential mechanism for regulating macrophage polarization. Activation of the mTOR pathway leads to macrophage polarization. Rapamycin, an autophagy inducer that inhibits the mTOR pathway, can stimulate the polarization of macrophages to the M1 phenotype [59], whereas CCL2 and IL-6 are potent autophagy-inducing factors that can trigger the polarization of macrophages to the M2 phenotype [60]. Additionally, dysfunction of autophagy in macrophages leads to their polarization toward the M1 phenotype. Therefore, induction of autophagy promotes the polarization of macrophages toward the M2 phenotype to minimize inflammation and enhance tissue regeneration [61]. In addition to regulating osteoblasts and osteoclasts, autophagy can regulate bone regeneration by modulating the immune microenvironment. The transformation of autophagy-activated macrophages from the proinflammatory M1 to the anti-inflammatory M2 phenotype can improve bone repair [62].

### Autophagy activation-based clearance of pathogens and infection treatment

Owing to the presence of bacteria, tissues are stimulated to produce cytokines and chemokines that recruit leukocytes to the infected tissue. The accumulation of leukocytes in the infected tissue interferes with the regeneration process and adversely affects the normal surrounding tissue [63]. Therefore, bacterial infection can activate various immune cells and induce inflammatory responses. During autophagy, pathogens are degraded, and the immune system of the host is activated, protecting the body from infectious diseases [64]. During bacterial infection, substrates are targeted through ubiquitination of bacteria or colocalization of ubiquitin with bacteria [65]. Autophagy receptors recognize ubiquitin and recruit bacteria to autophagosomes, leading to degradation of the bacterial cargo in lysosomes [66]. Mammalian cells can eliminate intracellular pathogens through autophagy, which enhances cell survival by contributing not only to the degradation of invaders such as bacteria, viruses, fungi, and parasites but also to the release of metabolites used by pathogens during infection [67]. Therefore, autophagy exerts efficient anti-infective and anti-inflammatory effects in the inflammatory phase, thus preventing tissue damage caused by excessive inflammation [68]. To date, several drugs that can induce autophagy have been successfully used in the treatment of infection [69]. AR-12, a small-molecule autophagy-inducing agent, can eliminate both intracellular and extracellular Francisella tularensis without causing cytotoxicity to the host [69]. Vitamin D inhibits infection caused by Mycobacterium tuberculosis and human immunodeficiency virus type 1 by inducing autophagy in macrophages [70]. Inhibition of bacterial infections is crucial for successful tissue regeneration [71].

### Autophagy activation promotes cell proliferation and differentiation

Regeneration of normal tissues requires coordination between the proliferation and differentiation of cells, which are essential processes for tissue regeneration [72].Lysosomal degradation pathways such as autophagy are necessary for cell proliferation and differentiation [73]. Activated autophagy regulates the stemness and differentiation of MSCs and maintains their survival under special conditions such as hyperglycemia, senescence, ROS accumulation, and hypoxia [74]. In addition, it plays a critical role in preserving MSCs after transplantation under high-stress conditions. Therefore, regulation of autophagy may play a crucial role in improving the wound-healing ability of MSCs in clinical settings [41]. Neural stem cells (NSCs) can undergo self-renewal and differentiation when regulated by Eva-1 homolog A (EVA1A) [75]. To maintain stem cell identity and function, protein turnover is especially important for eliminating the accumulation of unwanted proteins. As a clearance mechanism, autophagy eliminates damaged proteins and impaired organelles. Adult stem cells maintain their homeostasis through quiescence, self-renewal, and differentiation during autophagy. However, their survival and differentiation can be impaired owing to dysfunction of autophagy [73]. Therefore, autophagy is important for the proliferation and differentiation of stem cells, especially under stressful conditions [73].

### Autophagy activation inhibits apoptosis

As a stress adaptation pathway, autophagy contributes to cell survival during various types of stresses, such as nutrient and growth factor deprivation, disorders of the endoplasmic reticulum (ER), and protein aggregation [76]. Autophagy not only blocks the induction of apoptosis by inhibiting the activation of caspases related to apoptosis, thereby reducing cell damage but also helps to induce apoptosis [77]. Autophagy can be considered a nonapoptotic form of programmed cell death known as “type II” or “autophagic” cell death if it is overexpressed or uncontrollable under certain conditions [78]. Nonspecific degradation of large amounts of cytoplasmic proteins through autophagy may lead to cell death [79]. Several studies have demonstrated that autophagy promotes the survival and angiogenesis of human umbilical vein endothelial cells during re-epithelialization of wounds, thereby preventing apoptosis and oxidative stress injury and promoting the differentiation, proliferation, and migration of keratinocytes [80].

**Fig. 1:**
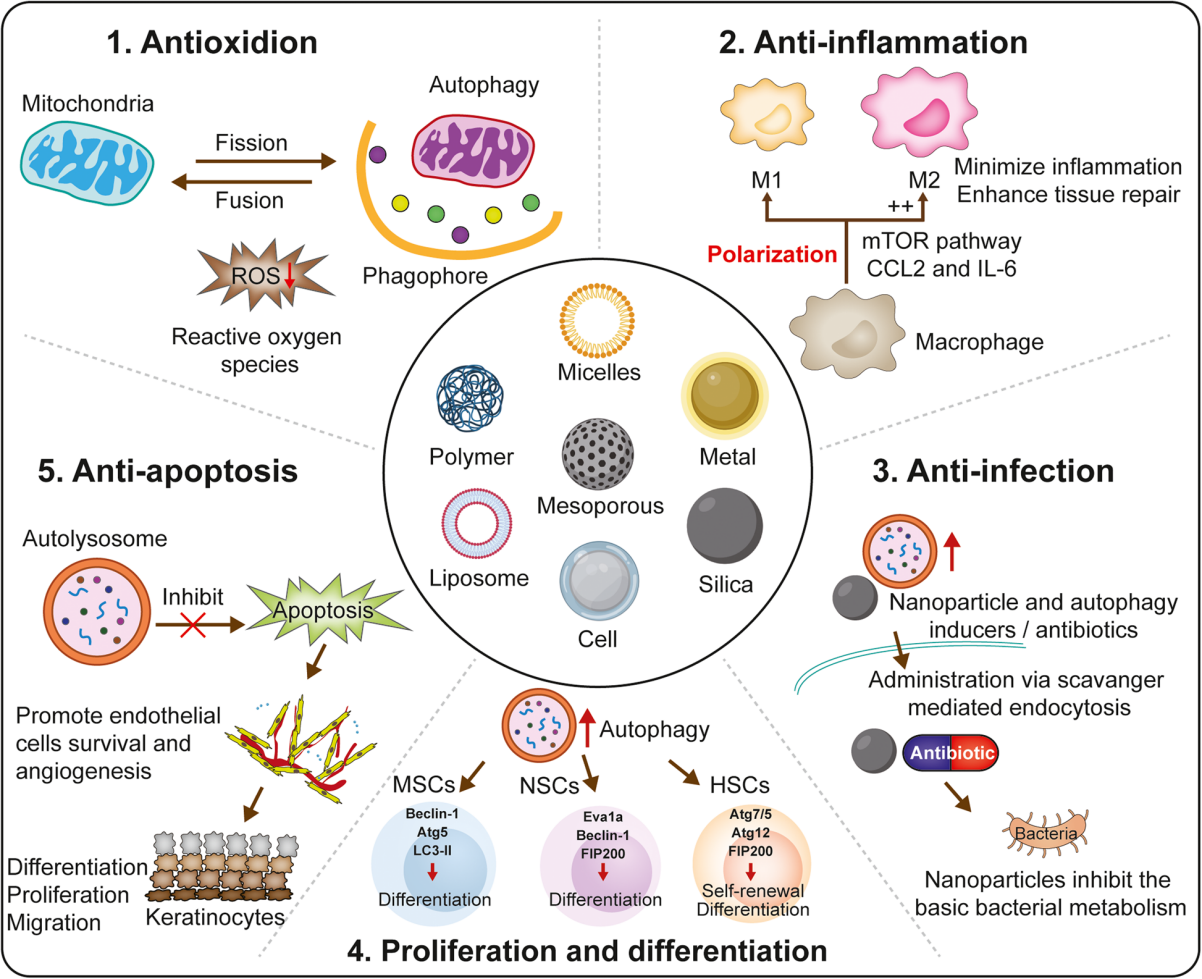
Mechanisms through which biomaterials regulate tissue regeneration via autophagy: Inhibition of oxidative stress, inflammation, infection, and apoptosis and promotion of cell proliferation and differentiation. (Created with adobe illustrate)

Therefore, autophagy is a double-edged sword because both excessive and limited autophagy can damage cellular homeostasis [79]. Excessive autophagy can induce cell death, whereas insufficiency or absence of autophagy can induce many diseases [81]. Similarly, autophagy can dramatically influence tissue regeneration, which is a highly complex and dynamic process. Biomaterials can affect various processes related to tissue regeneration by regulating autophagy. Biomaterials can promote and recruit stem or progenitor cells of the host by controlling the microenvironment, inducing native healing cascades, and enhancing cell differentiation and proliferation for in situ tissue regeneration [82]. Because autophagy plays a crucial role in tissue regeneration and either excessive or insufficient autophagy can result in cell death, efficient strategies are required for robust and precise control of autophagy. Therefore, therapeutic approaches based on biomaterial-regulated autophagy represent a novel strategy for tissue regeneration (Fig. 1).

## Autophagy and biomaterials

Owing to their unique physicochemical properties and good biocompatibility, biomaterials can interact with various cells after entering the human body and modulate autophagy through various mechanisms (Table 1).For example, metal nanomaterials, such as gold and silver nanoparticles, can directly trigger oxidative stress in cells, thereby activating autophagy [83]. Some carbon-based nanomaterials may lead to ER stress owing to the accumulation of misfolded and unfolded proteins, resulting in an increase in autophagy [84]. Moreover, some nanomaterials can block autophagic flux [85]. In addition to inducing autophagy, nanomaterials can be used as carriers to deliver autophagy regulators or genes targeting molecular components that could regulate autophagy [86]. Effective manipulation of nanomaterial-induced autophagy may expand the applications of nanomaterials in tissue regeneration. The pro-survival and pro-death autophagic effects of various nanomaterials can be effectively modulated via surface modification or artificial adaptation of the morphology, size, and composition. These modified nanomaterials can be used to induce autophagy to target specific cells to promote tissue regeneration. This section focuses on the effects of different types of biomaterials on autophagy and briefly describes their biological and medical applications.

### Metal nanomaterials

Owing to the excellent mechanical properties, chemical stability, and biocompatibility of metal implants, they are widely used inorthopedic and dental procedures to restore damaged tissues and structures. Therefore, the use of metal nanomaterials in tissue regeneration has attracted increasing attention. Metal nanomaterials, one of the most comprehensively investigated biomaterials, can trigger autophagy through various mechanisms, such as through mTOR signaling, production of ROS, and regulation of some ATGs [87].

**Table 1:** Regulation of autophagy in biological materials

Biomaterials	Target cells	Marker proteins/pathways (downregulation [↓]/upregulation [↑])	Autophagy regulation effect	Ref
Nanogold	NRK cells	LC3↑, p62↑	Blockage of autophagic flow, leading to lysosomal dysfunction	[88]
Fullerene	HeLa, MEF, MCF-7 cells	Atg5	Enhancement of autophagy	[89]
Single-walled carbon nanotubes	Primary glial cells from mice with Alzheimer’s disease	mTOR	Induction of autophagy and activation of lysosomes	[90]
Graphene oxide	HeLa, GFP-Htt(Q74)/PC12	P13K, MEK/ERK1	Induction of autophagy and removal of mutant proteins	[91]
Graphene Oxide Quantum Dots	GC2, TM4 cells	LC3↑/p62↑	Blockage of autophagic flow, leading to lysosomal dysfunction	[92]
Ag	MEF, HeLa	P13K	Induction of autophagy	[93]
Nano Ag	THP-1 Monocytes	LC3↑/p62↑	Blockage of autophagic flow, leading to lysosomal dysfunction	[94]
Dendritic macromolecules	A549 cells	AKT-TSC2-mTOR	Induction of autophagic cell death	[95]
Nano Si	L-02	EIF2AK3 ATF6UPR	Blockage of autophagic flow, leading to lysosomal dysfunction	[96]
cupric oxide	HUVEC	LC3↑/p62↑	Blockage of autophagic flow, leading to lysosomal dysfunction	[97]
Iron oxide	A549, IMR-90 cells	AKT-AMPK-mTOR	Induction of autophagy	[98]
Titanium Dioxide	HaCaT	LC3-II, Beclin 1, ATG5	Low concentration induces autophagy, high concentration blocks autophagy	[99]
DNATetrahedron	Chondrocytes	P13K/AKT/mTOR	Enhancement of cell autophagy	[100]

(Ag=silver;Si=Silicon; DNA=deoxyribonucleic acid; HUVEC = Human Umbilical Vein Endothelial Cells)

Gold nanoparticles (AuNPs) are associated with various physiological functions in cells [101]. It was observed that the accumulation of intracellular autophagosomes and elevated expression of the autophagy-associated protein LC3-II were caused by the blockage of autophagic flow after the entry of gold nanoparticles into cells [88]. Gold nanoparticles induce the accumulation of autophagosomes and the autophagic substrate p62 by blocking the fusion of autophagosomes and lysosomes. Furthermore, the cellular uptake of gold nanoparticles is particle-size dependent, with larger (50 nm) gold nanoparticles being more readily taken up into cells, leading to the accumulation of more autophagosomes compared with their smaller counterparts (10–25 nm) [88]. The gold nanoparticles engulfed by the cells accumulate in the lysosomes, alkalinizing them, leading to impaired lysosomal degradation and ultimately blocking autophagic flow. Studies have shown that AuNPs enhance the osteogenesis of periodontal ligament stem cell sheets by activating autophagy, which is upregulated by MAPLC3, and downregulation of the sequestosome 1/p62 pathway promotes osteogenic differentiation of the cell sheets [102]. The ability of AuNPs to promote osteogenic differentiation mainly depends on the activation of autophagy. Owing to their outstanding antibacterial properties, silver nanoparticles (AgNPs) are widely used in medical devices [103]. They can not only activate autophagy but also induce oxidative stress and cytotoxicity. Therefore, relevant biomaterials should be designed to modulate M2 macrophage polarization and regulate immunity for bone regeneration. For example, antibacterial AgNP-loaded TiO_2_ nanotubes (Ag@TiO_2_-NTs) promote bone tissue repair by inhibiting autophagy and downstream targets of the PI3K/Akt pathway and promoting polarization of macrophages to the M2 phenotype [104]. Titanium (Ti) is a common metallic biomaterial, and Ti implants with nanotopography can induce stronger autophagic responses, leading to the degradation of cytoplasmic Yes-associated protein (YAP). As YAP levels decrease, β-catenin is transported to the nucleus, where it accumulates to activate TCF/LEF transcription factors, leading to stronger osteogenesis [105].

In addition to metal compounds, metal-organic framework (MOF)-structured nanocatalysts can enhance ROS-induced oxidative damage by inhibiting autophagy, thereby synergistically treating diseases [106]. Iron-containing MOF nanocatalysts can be used as peroxidase mimics to catalyze the generation of specific hyperoxidative OH radicals, whereas chloroquine can be used to deacidify lysosomes and inhibit autophagy, thereby inhibiting the self-protection pathway under severe oxidative stress to promote tissue repair. Compared with the abovementioned three-dimensional MOF structures, two-dimensional (2D) MOF structures exhibit higher therapeutic potential owing to their typical planar topology, ultrathin thickness, and large specific surface area. A 2D organic framework based on transition metal nanomaterials can modulate autophagic responses, resulting in efficient chemical kinetics (CDT) for tissue regeneration [107]. In addition, some rare earth metal oxide crystals can induce neuronal autophagy by acting as “scavengers” that clear pathological protein aggregates. These crystals have potential clinical value for the treatment of neurodegenerative diseases [108]. In particular, selenomethionine (Se-Met) improves the initiation of autophagy through the AMPK-mTOR signaling pathway and enhances autophagic flux to promote tau clearance and improve cognitive impairment in Alzheimer’s disease (AD) in mice.

In conclusion, metal nanomaterials induce autophagy through various signaling pathways, and their effects on tissue regeneration are diverse. Therefore, understanding their role in inducing autophagy can not only help to understand the detailed molecular mechanisms underlying autophagy but also help to develop novel strategies for promoting tissue regeneration.

### Inorganic nonmetallic nanomaterials

Carbon-based nanomaterials, such as fullerenes and their derivatives, carbon dots, and graphene quantum dots, are a type of inorganic nanomaterial that are widely used in the field of biomedicine. They can induce autophagy through various mechanisms, including ROS generation in response to oxidative stress, mitochondrial dysfunction, and accumulation of polyubiquitinated proteins [108, 109].

Phagocytic nanomaterials can enhance the ability of cells to remove foreign substances by activating the autophagy-lysosome pathway (ALP), causing damage to downstream signaling pathways and blocking autophagic flux. The autophagy-inducing effects of fullerene derivatives can be greatly enhanced by modifying their surface functional groups [89]. Fullerene nanomaterials can lead to impairment of downstream signaling pathways of ALP, thereby blocking autophagic flux to enhance the cytotoxic effects of drugs. Fullerene C60, a tubular or spherical molecule composed of carbon atoms, can alleviate hyperglycemia-induced stress by regulating apoptosis and autophagy [110]. Owing to their biocompatibility, some inorganic materials can affect specific cellular functions; for example, graphene oxide (GO) can inhibit the viability and membrane integrity of MSCs through mitochondrial apoptosis and autophagy in a dose-dependent manner [111]. In addition to the ability of graphene to trigger autophagy, it may also induce diametrically opposed autophagic effects depending on its size, synthesis method, and surface functional groups. It has been observed that graphene oxide quantum dots [average diameter of (3.28 earth L16) nm] are capable of inhibiting lysosomal degradation by reducing the activity of histone B in GC-2 and TM4 cells, thereby blocking autophagic flow [92]. Conversely, a recent study involving mouse embryonic stem cells showed that graphene nanocolloids are capable of blocking autophagic flow by increasing lysosomal pH and membrane permeability while inhibiting the ensemble of autophagosomes and lysosomes [112]. These different findings illustrate the complexity of the use of nanomaterials to regulate autophagy. Therefore, it is necessary to analyze specific issues when using nanomaterials for autophagy regulation; generalizations should be avoided.

In addition to the abovementioned biomaterials, other inorganic molecules have also received substantial attention. A study reported that nanosized diamonds (NDs) and silica nanoparticles (SiO_2_-NPs) modulated autophagy in normal human facial skin fibroblasts (FSF1) by inducing a biphasic dose response [113]. NDs and SiO_2_-NPs at low concentrations (up to 0.5 mg/mL) played a beneficial role in increasing the proliferation and metabolic activity of FSF1 cells. Exposure of FSF1 cells to low concentrations of NDs and SiO_2_-NPs enhanced their in vitro wound healing capacity and delayed senescence during serial passage, which was evidenced by the maintenance of youthful morphological characteristics, reduced rates of telomere loss, and overall proliferative characteristics. Furthermore, selenium can inhibit the accumulation and toxic effects of arsenic in cells by inducing the mTOR/Akt signaling pathway [114]. Silica nanomaterials (SiNMs) are one of the most common engineering materials. Owing to their biological effects, the potential risk and toxicological effects of SiNMs have been a major focus of research [115]. SiNMs can induce ER stress and enhance autophagosome synthesis through ROS, thereby aggravating the accumulation of autophagosomes and eventually leading to disordered autophagy and cytotoxicity [96]. However, SiNMs play dual roles in cell survival and death by regulating autophagy. A study reported that bioactive silica nanoparticle formulations can drive the conversion of LC3β-I to LC3β-II, thereby increasing autophagosome formation, enhancing autophagy, and stimulating in vitro differentiation and mineralization of osteoblasts [116].

In conclusion, inorganic nonmetallic nanomaterials can regulate autophagy through various mechanisms. Owing to the unique physicochemical properties of inorganic nanomaterials, their detailed mechanism of action in regulating autophagy warrants further investigation. Because the regulatory role of some inorganic molecules in autophagy remains unclear, their safety and application should be further evaluated.

### Polymeric nanomaterials, hydrogels and microspheres

Polymeric nanomaterials are widely used in the clinical diagnosis and treatment of diseases owing to their diverse biological functions, high biocompatibility, and strong chemical stability. Several types of polymeric nanomaterials have been extensively investigated, such as hydrogels, microspheres, and nanocomposites. In recent years, hydrogels have attracted considerable attention owing to their simple preparation method and biocompatibility. Hydrogels have tunable mechanical and diffusion properties and present a cellular reactive fraction. In addition, they have excellent encapsulation capability, which can increase the stability and safety of cargo. Hydrogels can sense and respond to small changes in external stimuli, such as temperature, pH, ionicity, electric field, and magnetic field. Chitosan microspheres (CMs) encapsulating ivy (CM-SIN) can promote autophagy in chondrocytes and delay the progression of surgery-induced osteoarthritis [117]. Contamination-free sulfated zwitterionic poly (sulfobetaine methacrylate) (SBMA) hydrogels can improve pressure ulcer (PU) healing through rapid ECM remodeling. Additionally, SBMA hydrogels can inhibit the PI3K/Akt/mTOR signaling pathway to activate autophagy and reduce inflammation [118]. Understanding the relationship between autophagy and ECM remodeling may guide the design of biomaterial-based wound dressings for chronic wound healing. In addition to hydrogels, biomimetic polymer films have good morphological characteristics, thermal stability, and hydrophilicity. Composite nanofiber films composed of chitosan, PVP, and dihydroquercetin (DHQ) can facilitate wound healing by activating autophagy and increasing the expression of pankeratin, vascular endothelial growth factor (VEGF), and CD31 [28].

### Lipid nanomaterials

Lipid nanomaterials commonly comprise phospholipids, ionizable lipids, cholesterol, and PEGylated lipids, thereby functioning as novel and efficacious drug delivery systems [119]. Based on their structure, they can be classified as solid lipid nanoparticles, nanostructured lipid carriers, lipid drug conjugates, and polymer-lipid hybrid nanoparticles. Liposomes, which were first discovered by Bangham in 1965, are lipid-based nanomaterials that are easy to synthesize, biocompatible, and biodegradable and can be encapsulated with both hydrophilic and hydrophobic drugs [120]. In a previous study, we examined the effects of cationic liposomes on autophagic flux, autophagosome-lysosome fusion, and lysosome membrane permeabilization using liver cells [121]. The results revealed that cationic liposomes can induce permeabilization of the lysosomal membrane and inhibit late-phase autophagic flux, leading to the cytoplasmic release of proteinase B, mitochondrial dysfunction, and ROS production [121]. Additionally, cationic liposomes can elevate the pH of lysosomes to regulate autophagy in dendritic cells; however, anionic liposomes do not have these effects [122]. Lu et al. reported that lipid nanoparticles (LNs) can induce autophagy-lysosome signaling and neurovascular responses at least partially through an Atg5-dependent pathway [123]. Furthermore, lipid-based nanomaterials possess the capability to administer autophagy-regulating pharmaceuticals, thereby inducing or suppressing autophagy [124]. We have previously reported that the use of liposomes for the simultaneous delivery of salinomycin and the autophagy inhibitor chloroquine can increase the therapeutic efficacy of salinomycin by inhibiting the function of lysosomes [125].

### Cellular nanomaterials

Most types of cells secrete extracellular vesicles (EVs), which are crucial for intercellular communication. EVs are complex structures composed of multiple lipids, nucleic acids, and membrane proteins that have tissue-targeting ability. Therefore, they are often used as efficient carriers for the delivery of various drugs [126]. EVs are divided into four subgroups as follows: exosomes (30–150 nm in diameter), microvesicles (100–1000 nm in diameter), apoptotic bodies (100–5000 nm in diameter), and oncosomes (1–10 μm in diameter). EVs derived from MSCs (MSC-EVs) can deliver proteins and nucleic acids with regenerative functions to the injured site, triggering regenerative phenotypes and stimulating tissue regeneration. Additionally, the microRNA let-7a-5p present in MSC-EVs decreases apoptosis and increases autophagy in acute kidney injury (AKI). Therefore, MSC-EVs can be used to develop promising cell-free strategies for the treatment of AKI. To improve the stability and retention rate of EVs, EV-RGD hydrogels have been designed to regulate autophagy and promote the proliferation of renal tubular cells while retaining the biological properties of EVs [127]. In mouse models of osteoarthritis, infrapatellar fat pad (IPFP)-derived MSC-EVs can protect articular cartilage from damage by maintaining cartilage homeostasis and ameliorating gait abnormalities through mTOR inhibition regulated by miR-100-5p. This protective mechanism is similar to increasing the autophagy level of chondrocytes. Because IPFP is relatively easy to obtain from patients with osteoarthritis via arthroscopic surgery, exosomes derived from IPFP MSCs can be used to develop effective orthopedic therapeutic strategies. Several exosome-based biomimetic nanomaterials have been developed, such as Ti nanotubes functionalized with bone morphogenetic protein 2 (BMP2)/macrophage-derived exosomes, which can improve the biological function of Ti implants [128]. Ti nanotubes infused with BMP2/macrophage-derived exosomes can promote osteogenesis by activating autophagy and increasing the expression of alkaline phosphatase and BMP2 during osteogenic differentiation. Therefore, they can be considered an emerging bone regeneration material.

In conclusion, exosomes can regulate autophagy through various mechanisms, and exosome-based nanomaterials should be developed for clinical treatment. Additionally, because cellular biomaterials have the natural characteristics of cells, high biocompatibility, and low cytotoxicity, they should be extensively investigated for their role in tissue regeneration in the future.

### Other biomaterials

In addition to the abovementioned biomaterials, other biomaterials, such as quantum dots (QDs), can be used to modulate autophagy. QDs are semiconductors characterized by small size, variable surface chemistry, and the ability to absorb night-time ozone (NIR). Therefore, they are convenient tools for drug loading and photothermal treatment. QDs can be used for bioimaging because they are highly resistant to photobleaching, and QDs with different sizes or compositions can be excited to emit fluorescence at different wavelengths. Surface coating of QDs can regulate the induction of autophagy in body cells based on the properties of the coating substance. Peynshaert et al. compared two identical QDs with different surface coatings to determine the cytotoxicity and oxidative stress responses of the two coating substances, namely, 3-mercaptopropionic acid (3-MA) and PEG. They found that MPA-coated QDs were highly biocompatible and could be used for long-term cell tracking with minimal cytotoxicity. Mechanistically, MPA-coated QDs activated lysosomes and reduced ROS production, leading to activation of autophagy. However, PEGylated QDs induced significant autophagy dysfunction owing to increased ROS production and lysosomal impairment [129]. Self-assembling DNA nanomaterials have been extensively investigated in the field of biotechnology [130]. DNA nanomaterials are nontoxic and biocompatible and can precisely bind to and localize functional groups or other donor molecules at the nanoscale [130, 131].

As a novel autophagy regulator, biomaterials affect autophagy via a variety of mechanisms. Specifically, different biomaterials activate autophagy via different mechanisms owing to their different physicochemical properties. Furthermore, after entering cells, nanoparticles may regulate autophagy in three ways, namely, via oxidative stress (OS), the direct regulation of autophagic signaling pathways, such as the Akt/mTOR signaling pathway, and by altering the expression levels of autophagy-related genes or proteins [132]. OS, which is considered to be the main cause of nanoparticle-induced cytotoxicity, plays an important role in autophagy regulation [26]. Reactive oxygen species (ROS), a class of oxygen-containing chemically active molecules that are natural byproducts of normal oxygen metabolism, play a key role in cellular homeostasis, and their main cellular sources include the mitochondria, endoplasmic reticulum, peroxisomes, and NADPH oxidase complexes [133]. Inert nanomaterials, which cannot produce free radicals by themselves, can increase ROS production via interaction with the mitochondria, which is the most ROS-producing organelle in cells. Alternatively, metal or organic matter on the surface of nanomaterials can induce redox reactions. Additionally, excited-state electrons on the surface of the material can also lead to an increase in intracellular ROS levels. It has also been observed that ROS are involved in the mTOR signaling pathway, activating or inhibiting mTORC1 activity in a dose- and time-dependent manner; this in turn regulates autophagy. The degradation of nanoparticles in lysosomes can also directly induce ROS production. Lysosomes are considered a regular target of nanoparticle-induced cytotoxicity and autophagy [26]. The accumulation of nanoparticles in lysosomes, which leads to lysosomal swelling and the release of histone proteases, accompanied by elevated ROS levels, affects autophagy. Additionally, nanoparticles interact directly with the mTOR signaling pathway. It has also been reported that nanoparticle-mediated changes in mTOR activity are associated with changes in the mTORC1 activator AKT and its upstream target PI3K, as well as the regulation of the mTORC1 inhibitors AMPK and TSC [134]. During the intracellularization of nanoparticles, they may somehow affect the local PI3K/AKT recruitment/activation of the cell membrane, thus altering the ability of AKT to activate mTORC1 [134]. Furthermore, given that AKT can be activated by mTORC2, nanoparticle-mediated changes in AKT activity, at least in some cases, may be the result of secondary changes in mTORC2 regulation. The interaction of nanoparticles with lysosomes may also affect lysosome recruitment and mTORC1 activation. Additionally, nanoparticles are capable of activating nuclear translocation as well as the overexpression of TFEB, an important transcription factor of lysosomes, and enhancing the transcription of autophagy-associated genes (ATGs) as well as lysosomal genes, thereby promoting cellular autophagy [135].

In additon, the structure and arrangement patterns of the surfaces of biological materials at the micro and nano scales are called their topological properties. This property has implications for cell behavior: for example, grooves, ridges, and columns on the surface of biological materials can guide cell adhesion, proliferation, differentiation, and migration. Biomaterial topology and autophagy are two distinct but interconnected topics in the fields of biology and biomedical engineering. Studies have shown that biomaterials with specific topological patterns can influence cell behavior by modulating mechanical transformation pathways. This, in turn, can affect the level of autophagy within cells. For example, cells under mechanical stress or strain may up-regulate autophagy as a form of protection. Regulating autophagy through biomaterial topology may promote cell survival, reduce cellular senescence, and enhance tissue regeneration. Cells interacting with biomaterials may undergo autophagic processes to remove damaged or misfolded proteins or organelles caused by the foreign material. For example, optimized autophagy levels may support the maintenance and differentiation of stem cells in tissue engineering applications. Given that DNA nanostructures with engineered topological features and functions can facilitate the interaction between materials and cell surfaces, they can be used for biomedical applications. For example, ATG101 is an essential gene for initiating autophagy. ATG101 single-stranded antisense RNA-loaded DNA triangular nanoparticles (ssATG101-TNPs) can inhibit the expression of ATG101 to regulate autophagy and contribute to pulmonary vascular remodeling [136]. In addition, self-assembled DNA nanostructure-based delivery carriers can exert anti-inflammatory effects by promoting macrophage autophagy in the treatment of ALI [17]. In summary, biomaterial topology and autophagy are interconnected through their influence on cellular responses to biomaterials. Researchers are continually investigating how the design of biomaterials can impact autophagic processes to improve the performance and biocompatibility of medical devices and therapies.

**Fig. 2:**
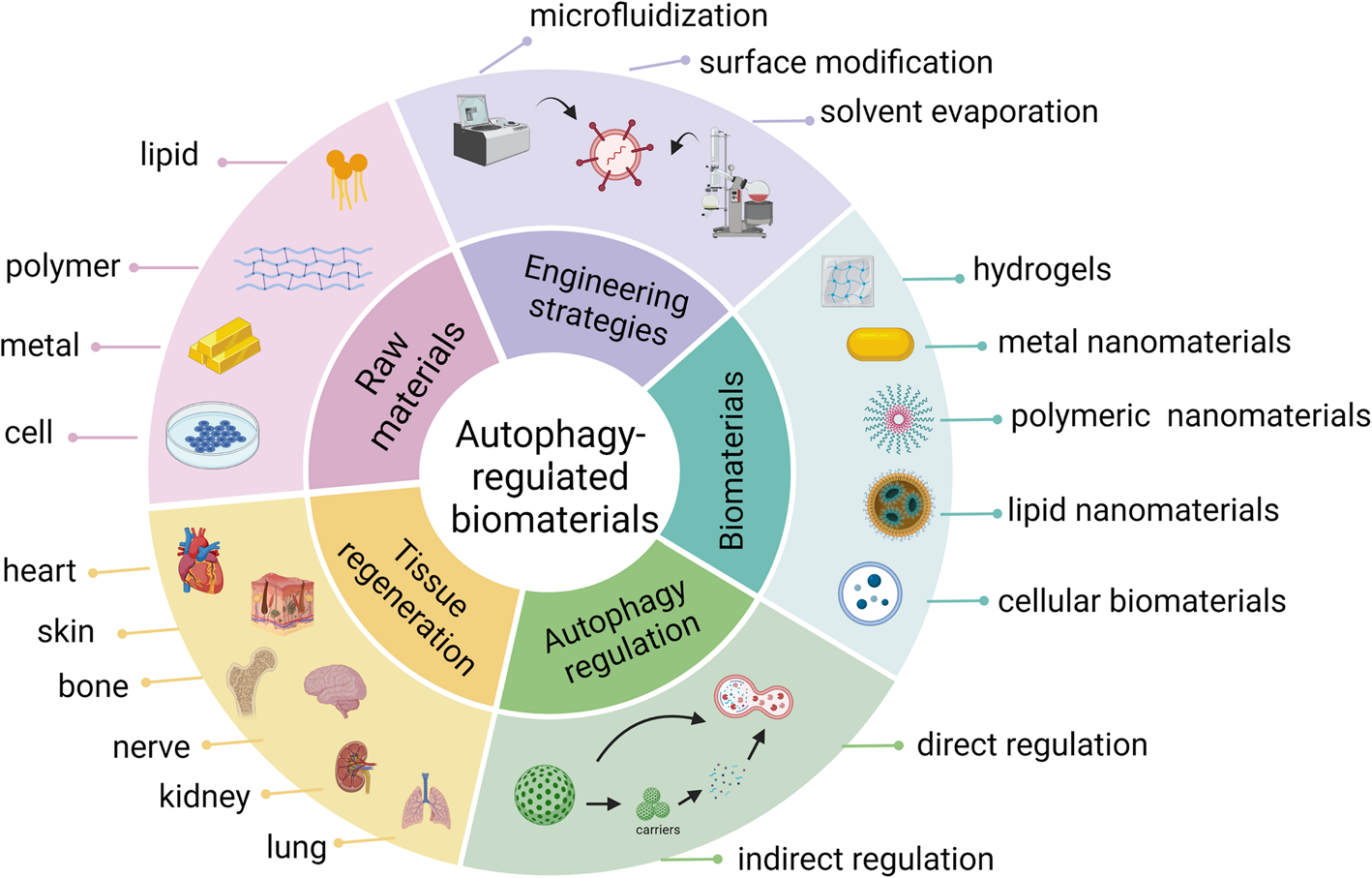
Summary of autophagy-modulating biomaterials in tissue regeneration. Various raw materials such as cells, metals, and polymers can be used to produce different types of biomaterials through engineering strategies including solvent evaporation, surface modification, and microfluidization. Biomaterials promote tissue and organ repair by regulating autophagy directly or indirectly. (Created with biorender.com)

In conclusion, cellular autophagy triggered by different biomaterials occurs via different phenomena. Even for the same biomaterial, the autophagic effect and mechanism triggered by biomaterials with different sizes, shapes and surface modification characteristics or even different target cell lines are different. These observations provide different models for the study of autophagy. Additionally, autophagy triggered by nanomaterials is two-sided. On the one hand, it can enhance the ability of cells to remove foreign substances. On the other hand, when excessive, it can also cause type II cell programmed death. Thus, the potential toxic effects of the nanomaterial on normal cells must be considered before its application. The use of biomaterials to induce autophagy at the cellular level has led to therapeutic advances in the medical field. To determine whether autophagy-modulating biomaterials are associated with tissue regeneration, studies on targeted tissue repair and regeneration in the bone, skin, nerve, heart, kidney, lung, and liver are summarized below (Table 2; Fig. 2).

**Table 2:** Regulation of biomaterial-based autophagy in tissue regeneration

Organ	Biomaterials	Target cell	Autophagy markers (downregulation [↓]/upregulation [↑])	Autophagy mechanism	Tissue repair markers (downregulation [↓]/upregulation [↑])	Biological effect	Ref
Bone	Sr-doped micro/nano rough titanium implants	BMSCs	For osteogenic differentiation of BMSCs: LC3II/LC3I↑, P62↓ and Beclin-1↑ For osteoclast differentiation: LC3II/LC3I↓, P62↑and Beclin-1↓	Not reported	For osteogenic differentiation of BMSCs: ALP↑, calcium nodules ↑ and Runx2, BMP-2, OCN and COL-1↑ For osteoclast differentiation: TRAF6, Ctsk and C-Fos↓	Upregulated autophagy and osteogenic differentiation in BMSCs, downregulated autophagy and differentiation in osteoclasts in vitro, improved implant osseointegration, decreased active osteoclast development and upregulated autophagy in bone tissue cells in vivo	[137]
	Sr-doped 45S5 bioglass	BMSCs	In the early phase: LC3II/LC3I↑ and Beclin-1↑ In the late phase: LC3II/LC3I↓ and Beclin-1↓	AKT/mTOR	ALP and calcium nodules ↑	Improved autophagy and promoted osteogenic differentiation of OVX-BMSCs and bone regeneration in osteoporotic bone defects	[138]
	Resveratrol and angiogenin-2 combined with PEGDA/TCS hydrogels	BMSCs and HUVECs	LC3II/LC3I↑, P62↓and Beclin-1↑	Not reported	Ki67 ↑, CD31↑, ALP ↑, calcium nodules ↑and Runx2 and OPN↑	Promoted BMSC differentiation and vascularization and tissue repair in the tibial defect through autophagy	[139]
	Silver nanoparticle-loaded TiO_2_ nanotubes	RAW264.7 and MC3T3-E1 cells	LC3II/LC3I ↑and Beclin-1 ↑	PI3K/AKT and GLUT1	ALP, RUNX2, OCN and OPG ↑	Activated autophagy, regulated bone immunity and promoted osteogenesis	[104]
	Gold nanoparticles	Periodontal ligament stem cells (PDLSCs)	LC3II↑and P62↓	Not reported	ALP, calcium nodules↑; RUNX2, OCN and COL-1↑	Enhanced osteogenesis of PDLSC sheets by activating autophagy	[102]
	Dicalcium silicate nanoparticles	BMSCs	LC3II/LC3I↑, P62↓and Beclin-1↑	mTOR/ULK1 and WNT/β-catenin	Calcium nodules ↑ BMP2, UNX2 and OSX↑	Enhanced bone formation and osteogenic differentiation by activating autophagy	[140]
	Nanosized alumina particles and bortezomib	MG-63 cells	LC3↑	Nf-κB	OPG↑	Activated autophagy and inhibited apoptosis	[141]
	Titanium implants with nanotopography	MC3T3-E1 cells	LC3II/LC3I↑ and P62↓	YAP and β-catenin	ALP and Osx ↑	Activated autophagy and promoted osteogenesis	[18]
	Solid silica nanoparticles (SSN)	BMSCs	LC3II ↑	ERK1/2 and AKT/mTOR	ALP, COLI, OPN, OPG, RUNX2 and OCN↑	Improved osteogenic differentiation by increasing autophagy	[142]
	Nanohydroxyapatite	MC3T3-E1 cells	LC3II/LC3I↑	m-TOR	ALP, BMP2, OSC, BSP, BMP2 and RUNX2↑	Modulated osteoblast differentiation by mediating autophagy in a dose-dependent manner	[143]
	Sinomenine encapsulated within chitosan microspheres and photo-crosslinked GelMA hydrogels	Mouse chondrocytes	LC3↑	Not reported	MMP13 and ADAMT-5 ↓, COL2A1 and AGGRECAN↑and safranin-O staining ↑	Retarded the progression of surgically induced OA and ameliorated cartilage matrix degradation at least partially through autophagy	[117]
	miR-100-5p-abundant exosomes	MSCs	LC3II↑and P62↓	mTOR	Collagen II↑, MMP 13 and ADAMTS 5↓and safranin-O staining↑	Protected articular cartilage from damage and ameliorated gait abnormality in mice with OA	[144]
	Kartogenin/reduced graphene oxide@gelatin	ADSCs	LC3II/LC3I↑, Beclin-1↑and ULK1↑	Not reported	Sox-9↑, Col II ↑, alcian blue staining ↑and toluidine blue staining↑	Promoted chondrogenesis synergistically by modulating autophagy	[145]
Skin	Metal-organic frameworks	Mouse embryonic fibroblasts	LC3II/LC3I↑, P62↓, Beclin-1↑and ATG5↑	mTOR	Cell apoptosis↓	Reduced ROS production and induced cytoprotective autophagy	[146]
	MSCs	Endothelial progenitor cells	LC3II/LC3I↑, Beclin-1↑and ATG7↑	ERK	wound size↓, CD31 ↑, tube formation↑and VEGF↑	Enhanced full-layer cutaneous wound healing and promoted the paracrine secretion of VEGF in MSCs	[147]
	PDGF-PLGA hydrogels	HUVECs and 3T3 cells	LC3II/LC3I↓	Not reported	Migration rate, granulation tissue formation and collagen deposition↑	Promoted the proliferation and migration of cells and accelerated the closure of full-thickness excision wounds by inhibiting autophagy	[34]
	Sulfobetaine methacrylate hydrogels		LC3II↑and P62↓	PI3K/Akt/mTOR	Wound size↓, granulation tissue ↑, collagen deposition↑, collagenI/III ↑, CD68↓ and CD206↑ Fibronectin ↑, laminin↑ and MMP-2↓	Improved pressure ulcer healing by promoting ECM reconstruction by inducing autophagy	[118]
	Chitosan/PVP/dihydroquercetin nanocomposite films	Hacat cells	LC3II/LC3I↑, Beclin-1↑, p62↓, ATG5↑and ATG7↑	PI3K/Akt/mTOR	Wound size↓, granulation tissue ↑, collagen deposition↑ VEGF, CD31 and pankeratin↑	Promoted wound healing by activating autophagy	[28]
Nerve	Polycaprolactone neural guide conduit loaded with melatonin	Sciatic nerve cells	LC3A/B↑; Beclin1 and LC3I↑and ATG3, ATG5 and ATG7↑	Not reported	Sciatic function index↑, nerve conducting velocity↑, regenerated axon area↑ c-caspase↓	Enhanced autophagy, reduced apoptosis and restored proliferation of neurons	[148]
	Single-walled carbon nanotubes	CRND8 glial cells	p-ULK1↓, LC3↑and p62↓	mTOR	Lysosome number↑ active CatD↑	Induced autophagy, restored lysosomal function and facilitated the elimination of autophagic substrates	[90]
	Chitosan-based nanosweeper combined with PEGylated-GKLVFF and Beclin-1 peptide	N2a cells and hippocampal neurons	LC3II↑, p62↑and LC3↑	Not reported	Soluble and insoluble Aβ42↓and escape latencies↓	Induced autophagy and Aβ clearance, increased cell viability and rescued memory deficits	[149]
	Nanosized polyethylene glycol loaded with a curcumin analog	N2a cells	LC3↑	Not reported	α-syn↓	Induced autophagy and clearance of α-syn	[150]
	Gold nanoclusters with dihydrolipoic acid	BV2 cells	LC3II/LC3I ↑and P62 ↑	Not reported	Arg-1 and CD206↑, iNOS↓NOX4 and ROS production↓	Induced autophagy with the polarization of macrophages to the M2 phenotype and reduced oxidative stress caused by ROS	[151]
	Graphene oxide-coated electrospun nanofibers loaded with methylene blue	Neural progenitor cells (NPCs)	LC3II↑	Not reported	G0/G1 phase cells↑ p-tau S262↓	NPCs entered the quiescence phase, and degeneration of p-tau was increased	[152]
	Neuron-derived exosomes loaded with miR-21-5p	HT-22 neurons	LC3, P62 and Beclin-1↓	Rab11a	caspase-3 and Bcl-2↓	Inhibited autophagy and attenuated nerve injury	[153]
	Europium hydroxide [EuIII(OH)_3_] nanorods	Neuro 2a, PC12 and HeLa cells	LC3II/LC3I ↑and P62↓	Not reported	GFP-Htt (Q74)↓	Enhanced the clearance of the huntingtin protein	[154]
Heart	Magnetic mesoporous silica-coated Fe_3_O_4_ nanoparticles loaded with N-acetylcysteine	Cardiomyocytes	p62↓ LC3II↓	Not reported	LDH activity↓, MMP↑, caspase-3 and Bax↓; Bcl-2↑ MDA, 8-OHDG and 8-iso-PGF2α↓and GSH, CAT, GSH-Px and SOD↑	Reduced apoptosis and ROS generation induced by Fe_3_O_4_ NPs by reducing autophagy	[155]
	Perfluorocarbon particles loaded with rapamycin	Cardiomyocytes	p62 ↓ BNIP3 and LC3II ↑	mTOR	LVEF↑	Increased cardiac contractile performance and LVEF	[156]
Kidney	Kidney injury molecule-1-Res NPs	Human proximal tubular epithelial cell line HK-2	LC3II↑, Beclin-1↑and p62↓	AMPK mTOR	Blood urea nitrogen↓, creatinine↓, NLRP3↓ IL-1β↓	Suppressed the NLRP3 inflammasome by enhancing autophagy and ameliorated chronic kidney disease	[27]
	Extracellular vesicle (EV)-RGD (Arg-Gly-Asp) hydrogels	HK-2 cells and mouse macrophages RAW263.7	LC3B↑	miRNA let-7a-5p	SCr and BUN↓, Kim-1↓	Elevated cell autophagy, improved renal function, decreased tubular injury and ameliorated histopathological impairments	[127]
	Magnetic Fe_3_O_4_ nanoparticles with albumin (Fe_3_O_4_@BSA)		Autophagosome↑	Rab7	MMP-2↑, α-SMA↓, ALB, BUN and Scr↓, urinary protein and NAG↓ collagen accumulation↓	Attenuated renal tubular injury and tubulointerstitial fibrosis	[157]
	Ceria-zirconia nanoparticles	HK‑2 podocytes	LC3II/LC3I↑	AKT/mTOR	ROS levels↓, cell apoptosis↓TGFβ1, fibronectin and α-SMA↓	Reduced intracellular globotriaosylceramide accumulation and alleviated kidney injury	[158]
Lung	Rapamycin (mTOR) siRNA-loaded DNA nanotubes (DNA-NTs)	Pulmonary arterial smooth muscle cells (PASMCs)	LC3II/LC3I↑	mTOR	Cell proliferation↓	Efficiently delivered siRNA to PASMCs, leading to strong autophagy induction, and inhibited the proliferation of PASMCs	[159]
	mTOR siRNA-loaded spermidine/DNA tetrahedron nanoplatform	Bone marrow-derived macrophages (BMDMs)	LC3B↑and P62↓	mTOR	M1 polarization↓ M2 polarization↑	Exerted anti-inflammatory effects by promoting autophagy and phenotypic transition of macrophages and relieved acute lung injury	[17]
Liver	Nifedipine-loaded nanoparticles composed of poly(lactic-co-glycolic acid) (PLGA)	HepG2 cells	P62 and ubiquitin↓	Not reported	Oil red O staining ↓, liver mass↓ average adipocyte area of epididymal white adipose↓	Improved insulin resistance and hepatic steatosis through autophagy	[160]
	Superparamagnetic iron oxide nanoparticles (SPIONs)	RAW 264.7 cells	LC3B II and Beclin-1 ↑	Cav1-Notch1/HES1	AST ↓, ALT↓	Promoted IL-10 expression and inhibited inflammation by activating autophagy in models of LPS-induced sepsis and liver injury	[161]

## The application of autophagy-modulating biomaterials in tissue regeneration

### Autophagy-based biomaterials for bone and cartilage regeneration

**Fig. 3:**
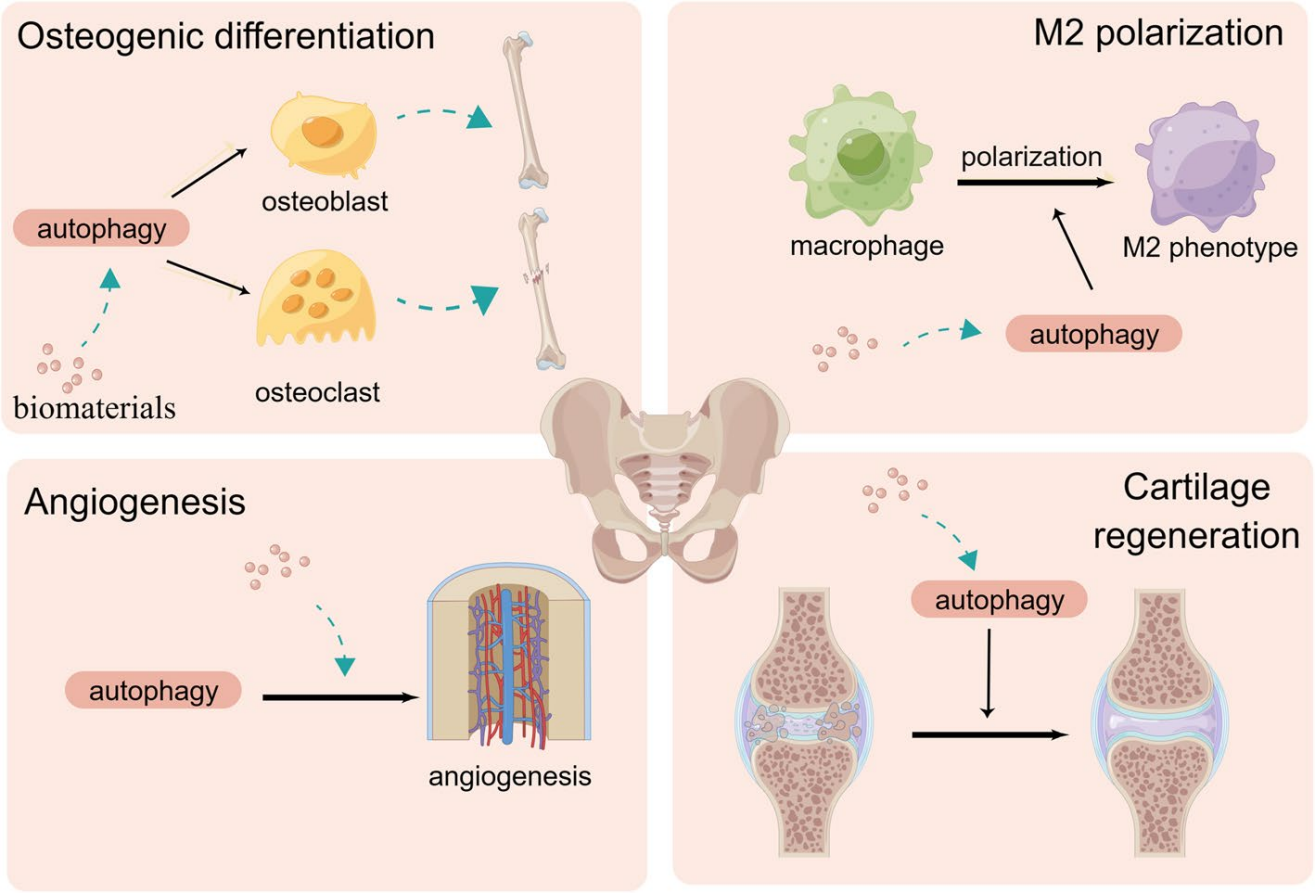
Schematic diagram of the regulation of bone and cartilage regeneration through autophagy. Autophagy promotes bone regeneration by modulating osteogenic differentiation, inducing M2 polarization, promoting angiogenesis, and improving cartilage regeneration. (Created with figdraw.com)

Bone is one of the most dynamic organs in the body and is continuously renewed throughout life. Bone remodeling includes both formation and resorption, which collectively maintain bone homeostasis. Bone loss is caused by many physiological and pathological conditions, such as aging, exposure to chemicals, fractures, and diseases such as osteoporosis [162]. Reconstructing large bone defects can be challenging in both orthopedic and dental treatments. Bone is primarily composed of osteoblasts, osteocytes, and osteoclasts. Osteoblasts secrete the organic matrix of bone (osteoid) and participate in its mineralization. During their encasement in bones, osteoblasts undergo complete differentiation to become osteocytes, which orchestrate bone remodeling. Osteoclasts from hematopoietic stem cells continually degrade and absorb the bone matrix surrounding them. Bone mass is affected by the reciprocal action of osteoclasts. Cartilage is a connective tissue found mainly in the throat, respiratory tract, external ear, and surface of the bone in the joint. It is produced by chondrocytes, and its primary function is to maintain the normal structure and function of bone. Chondrocyte destruction decreases the cartilage matrix and affects nutrition and metabolism in the cartilage, which may lead to osteoarthritis. Several recent studies have reported the applications of biomaterials in bone and cartilage regeneration [163, 164] Autophagy plays a key role in maintaining bone and chondrocyte homeostasis by regulating cell survival, cell differentiation, and stress responses [165]. Wang et al. demonstrated that nanohydroxyapatite modulates osteoblast differentiation by mediating autophagy in a dose-dependent manner [143]. Autophagy provides energy to starving cells and removes dysfunctional or damaged proteins and organelles [165, 166]. Therefore, autophagy is important for bone and cartilage regeneration owing to its role in the differentiation and polarization of immune cells and other bone cells (Fig. 3). On the one hand, as a cellular survival mechanism, autophagy regulates the balance of bone and cartilage remodeling by directly participating in the differentiation of osteoclasts, osteoblasts, and chondrocytes. On the other hand, autophagy may influence bone and cartilage regeneration by regulating immune cell responses, which subsequently influence the osteogenic and chondrogenic microenvironment. Recent studies have demonstrated that autophagy-modulating biomaterials can promote the treatment of bone-associated diseases by regulating osteoblasts and chondrocytes. The role of autophagy-modulating biomaterials in promoting bone regeneration is discussed in the following sections (Fig. 3).

### Bone regeneration

Osteocytes constitute the majority of cells in bones. They are formed via terminal differentiation of osteoblasts engulfed by ECM during bone formation. Osteoblasts form new bone from pluripotent MSCs through a process known as osteogenesis. They synthesize and secrete numerous collagen type I molecules, which help to mineralize the bone matrix. Osteoclasts, the third type of bone cell, are large multinucleated cells capable of bone resorption. Bone cells can survive under adverse conditions through an appropriate level of autophagy [162]. Autophagy plays an important role in the differentiation of preosteoblasts and osteoblasts to osteoclasts and the genesis and function of osteoclasts [167]. The role of autophagy in alleviating bone-related diseases, including osteoporosis, has been extensively investigated [168]. Autophagy is strongly associated with bone regeneration and homeostasis maintenance. According to Nuschke et al., autophagy is required for maintaining the stemness and differentiation of bone marrow-derived MSCs (BMSCs) [169]. Additionally, Wan et al. reported that autophagy induces osteogenesis in vivo and is promoted by the AKT/mTOR signaling pathway [170].

Owing to their excellent mechanical properties, chemical stability, and biocompatibility, metal implants are widely used in orthopedic and dental surgeries to restore damaged tissue and structure [171]. The nanosized surface of metal biomaterials can adequately interfere with intracellular autophagy regulation. Compared with flat surfaces, nanotubes can strongly induce mTOR-independent autophagy in osteoblasts [172]. Ti implants with nanotopography can modulate osteogenic differentiation associated with increased nuclear translocation of β-catenin and ALP-mediated degradation of cytoplasmic YAP [18]. It is important to note that Ti particles are involved in particle-induced diseases. Therefore, metal materials, including particle-reinforced composite materials, scaffolds for tissue engineering, and drug delivery materials, should be used with caution. Studies have also shown that Ti particles can induce apoptosis by enhancing autophagy related to the PI3K/Akt signaling pathway; this can potentially impair local innate immunity in periprosthetic tissues, thereby increasing the risk of late-onset periprosthetic joint infection [173]. In an experimental mouse model of calvarial bone resorption induced by Ti particles, nanosized alumina prevented the activation of the autophagy marker LC3, inflammation, and osteolysis induced by Ti particles [141]. Therefore, implant design should be cautiously decided upon when Ti is inevitably used. Studies have demonstrated that Sr promotes osseointegration. Wang et al. developed Sr-doped micro/nano rough Ti implants via hydrothermal treatment (SLA + Sr), which promoted osteogenic differentiation and inhibited osteoclast differentiation by regulating autophagy [137]. Sr may exert different physiological effects based on the disease stage. Zhang et al. designed Sr-doped 45S5 bioglass (Sr/45S5), which markedly enhanced bone regeneration by improving autophagy during early-stage disease and activating the Akt/mTOR signaling pathway during late-stage disease [138]. Furthermore, the therapeutic effects of metal materials depend on their size. Compared with 13-nm AuNPs, 45-nm AuNPs significantly promoted the osteogenic differentiation of periodontal ligament stem cells by upregulating bone-related protein expression and mineralization (see Fig. 4a-c in Ref. [102]). BMP2/macrophage-derived exosomes can improve the biofunctionality of Ti implants [174]. Ti nanotubes functionalized with BMP2/macrophage-derived exosomes can significantly enhance the expression of early osteoblast differentiation markers (alkaline phosphatase and BMP2) by activating autophagy during osteogenic differentiation. Therefore, autophagy-modulating biomaterials can regulate the differentiation and mineralization of bone cells during bone regeneration. Given that the surface, size, or combined use of metal biomaterials can influence therapeutic outcomes, special attention should be given to the design, fabrication, and application of biomaterials for bone regeneration.

For new bone formation, biomaterials provide locations for MSC proliferation and differentiation. For example, it is believed that hydrogels are composed of hydrophilic polymers that can mimic bone tissue extracellular matrix and thereby significantly support cell growth and osteogenesis [175]. Owing to severely hypoxic microenvironments, hydrogels used for bone tissue engineering fail to repair bone defects. Fan et al. fabricated small-aperture PEGDA/TCS hydrogels containing resveratrol and angiopoietin-2 (ANG2) [139], which were cocultured with BMSCs in vitro and implanted into rats with a large-segment tibial bone defect. The hydrogels induced angiogenesis at the defect site through autophagy in a hypoxic environment, thereby promoting bone tissue formation (see Fig. 4d in Ref. [139]). In addition to directly contributing to osteogenesis, biomaterials can promote immunoregulation in tissue engineering strategies for treating bone defects. Biomaterials implanted into the body often fail to regenerate bone because of the host immune response, especially macrophage-related inflammation, which is important for bone healing [176]. Therefore, designing macrophage-related immunomodulatory biomaterials is a promising strategy for enhancing osseointegration. Chen et al. used electrochemical anodization to develop antibacterial TiO_2_ nanotubes with AgNP-loaded surfaces (Ag@TiO_2_-NTs) [104]. The nanotubes induced macrophage polarization toward the M2 phenotype and created a suitable osteoimmune microenvironment by inhibiting PI3K/Akt and activating autophagy to promote bone healing. Multifunctional cells, ECM, and numerous related factors play a role in treating bone disorders. Therefore, autophagy can provide a suitable environment for bone regeneration by maintaining cellular homeostasis.

### Cartilage regeneration

The aneural and avascular nature of cartilage severely limits its capacity for self-regeneration. Osteoarthritis is a classical chronic aging disorder that destroys cartilage and synovial membranes. Autophagy can improve the viability and function of chondrocytes, thereby delaying the progression of cartilage-related disorders [177]. Rapamycin can act as an autophagy activator owing to its ability to limit the inhibitory effects of mTOR on autophagy via the ULK-Atg13-FIP200 complex [178]. Activation of autophagy via rapamycin has been shown to reduce the severity of osteoarthritis in experimental models [179]. Nevertheless, the development of specialized drug delivery systems to address the hydrophobic nature and limited bioavailability of rapamycin presents a formidable task. To address this concern, Pape et al. developed biodegradable rapamycin-loaded poly(lactic-co-glycolic acid) (PLGA) nanoparticles using the emulsion/evaporation method [180]. These nanoparticles were found to be suitable for intra-articular administration owing to their size, high encapsulation efficiency, and sustained release of rapamycin, resulting in positive therapeutic effects on local articular joint inflammation in an animal model of osteoarthritis.

As a novel nanomaterial for cartilage tissue engineering, GO has been extensively examined both in vivo and in vitro for its hydrophilicity, functionality, osteoinductive properties, biocompatibility, and toxicity. The toxicity of GO depends on various factors, including lateral size, surface structure, functionalization, charge, the presence of impurities, aggregation, and the corona effect [181]. Owing to its aromatic structure, GO can promote the adhesion, proliferation, and differentiation of cells [182]. In addition, it can be used for cartilage regeneration because of its mechanical properties [183]. Additionally, GO can modulate the viability and membrane integrity of MSCs through mitochondrial apoptosis and autophagy in a dose-dependent manner [111]. As a biocompatible material, reduced GO can deliver bioactive drugs to stimulate stem cell-directed differentiation and influence specific cellular functions. Jiao et al. fabricated biodegradable gelatin-reduced GO (rGO@Ge) and used it as a biocompatible carrier to deliver kartogenin to adipose-derived MSCs to promote chondrogenesis and augment the pro-chondrogenic effects of the drug [145].

MSCs exhibit promising potential for cartilage regeneration [184]. Several studies have demonstrated that exosomes derived from MSCs inhibit the progression of osteoarthritis by maintaining chondrocyte homeostasis and ameliorating pathological severity [185]. IPFP-MSC-derived exosomes can maintain cartilage homeostasis and reduce gait abnormalities by protecting articular cartilage from damage in mice with osteoarthritis. The underlying therapeutic mechanism may involve inhibition of mTOR and activation of autophagy mediated by miR100-5p present in MSC-derived exosomes [144].

Active components found in traditional Chinese medicinal extracts can perform many biological functions related to autophagy in cartilage regeneration [117, 186]. Cordycepin can prevent cartilage degradation partly by activating autophagy, and hydrogels prepared using the combination of chitosan microsphere-cordycepin and photocrosslinked hyaluronic acid methacrylate (HAMA) can delay the progression of surgically induced osteoarthritis and alleviate the degradation of the cartilage matrix by inducing autophagy [186]. Similarly, sinomenine has been shown to alleviate IL1-β-induced degradation of the cartilage matrix partially by triggering autophagy in chondrocytes and an ex vivo model. Hydrogels prepared using the combination of chitosan microsphere-sinomenine and photocrosslinked gelatin methacrylate (GelMA) can delay the progression of surgically induced osteoarthritis and alleviate the degradation of the cartilage matrix in vivo by enhancing autophagy [117]. Therefore, the independent use of certain drugs can promote cartilage regeneration via autophagy, and the combined use of these drugs with some polymers can enhance their therapeutic efficacy in cartilage-related diseases.

**Fig. 4:**
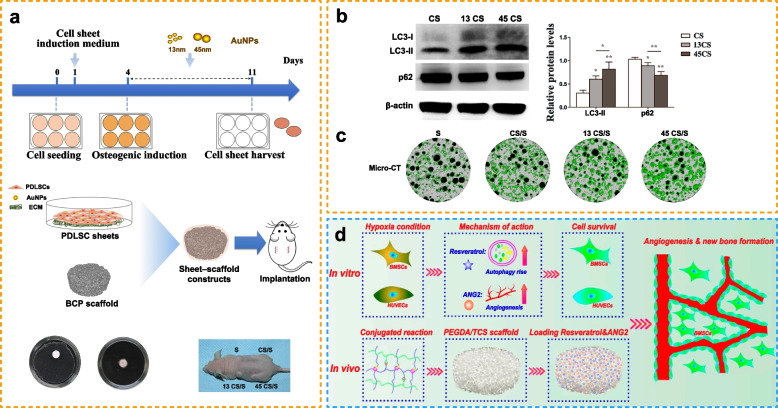
Autophagy-modulating biomaterials promote bone regeneration. **a** Design scheme and fabrication. **b**) Western blotting for quantification of LC3 and p62. **c** Representative micro-CT images for examining bone regeneration promoted by gold nanoparticles (Reproduced with permission [102]; Copyright 2021, Dove Medical Press Ltd.). **d** Schematic illustration of the synergistic therapeutic effects of Res and ANG2 on bone defects under hypoxic conditions (Reproduced with permission [139]; Copyright 2021, Frontiers Media S.A.

### Autophagy-based biomaterials for skin regeneration

The skin serves as a barrier between external elements and internal organs and is capable of excellent regeneration. Any injuries or cuts on the skin can be healed through a highly orchestrated sequence of physiological processes. Cutaneous wound healing is a complex, multistep process that precisely controls the proliferation and migration of cells, deposition of ECM, angiogenesis, and vascular remodeling (see Fig. 5 in Ref. [80]). Refractory wounds, such as diabetic skin ulcers, can cause serious health problems [187, 188]. Previous studies have validated the role of autophagy in various phases of wound healing. In the inflammatory phase of wound healing, autophagy exerts anti-infective and anti-inflammatory effects, thus protecting tissues from damage caused by excessive inflammation [189]. In the proliferative phase of wound healing, local hypoxia promotes autophagy, which plays a critical role in inhibiting apoptosis and oxidative stress and promoting cell survival. Autophagy can stimulate wound re-epithelialization by promoting angiogenesis in vascular endothelial cells (ECs) and the differentiation, proliferation, and migration of keratinocytes [190]. During tissue remodeling, fibroblast autophagy contributes to hypertrophic scar formation [37, 191]. Because skin wound healing involves the activation of multiple factors, understanding autophagy, the complex process of wound healing, and the molecular mechanisms underlying autophagy regulation can help to develop new strategies for the clinical treatment of wounds. Biomaterials are promising tools for promoting skin wound healing. Zarei and Soleimaninejad summarized biomaterials that can promote cutaneous wound healing, such as nanofibrous chitin and chitosan [192]. Additionally, studies have demonstrated the interaction between biomaterials and cells. Autophagy plays a crucial role in cellular functions such as phagocytosis, biomaterial clearance, cell differentiation, and stress responses [193].

**Fig. 5:**
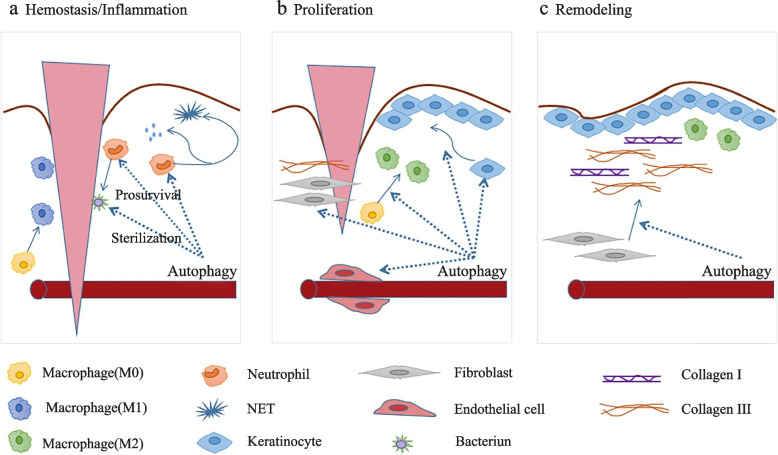
Role of autophagy in cutaneous wound healing. Wound healing is regulated by autophagy in three stages: proliferation, hemostasis, and remodeling. Autophagy enhances the survival, proliferation, and migration of neutrophils, macrophages, endothelial cells, keratinocytes, and fibroblasts, which are essential processes for the biological functions of cells and aid in wound healing. NET, neutrophil extracellular trap. (Reproduced with permission [80]; Copyright 2022, Oxford University Press)

Fibroblasts contribute to wound healing through several processes, such as degradation of the fibrin clot, formation of new ECM and collagen structures to support other cells associated with effective wound healing, and contraction of the wound [194]. Li et al. designed and synthesized nonfouling zwitterionic sulfated SBMA hydrogels that improved pressure ulcer healing with rapid reconstruction of ECM by inducing the expression of laminin and fibronectin and inhibiting MMP-2 [118]. According to the results of a subsequent study, MMP-2 expression is decreased following zwitterionic SBMA hydrogel treatment because dysfunctional PI3K/Akt/mTOR signaling directly activates autophagy (see Fig. 6-c in Ref. [118]). Furthermore, the effect of biomaterials is directly related to their dose. Treatment with low concentrations of NDs and SiO2-NPs can improve their wound healing ability and delay aging during serial passage [113]. NP treatment activates Nrf2- and FOXO3A-based cellular stress responses, which are associated with autophagy [195]. In addition, the utilization of micromaterial- and nanomaterial-based delivery systems has the potential to significantly enhance the efficacy of drug delivery, thereby prolonging the duration of drug circulation for the purpose of tissue regeneration. Xu et al. designed and synthesized PLGA microspheres to deliver platelet-derived growth factor (PDGF) [34]. Hydrogels prepared using PDGF-PLGA microspheres had excellent biocompatibility, supported the growth and migration of HUVECs and 3T3 cells, and accelerated wound healing by inhibiting autophagy. Therefore, it is necessary to investigate the role of autophagy in full-thickness skin damage. The abovementioned studies demonstrate the effects of biomaterials on autophagy and elucidate the underlying mechanisms, which can help to understand the important role of autophagy in both the proliferative and remodeling phases of wound healing and accelerate research into the autophagy-modulating properties of biomaterials.

Wound healing occurs in multiple steps at the cellular level, including inflammation, re-epithelialization, and maturation. Keratinocytes play a significant role in re-epithelialization. They proliferate and migrate into the wound, resulting in the formation of new epithelium. Several studies have shown that inhibition of autophagy in keratinocytes impairs skin regeneration in mice [190, 196]. In a recent study, mice were genetically modified by deleting ATG5 or ATG7 to inhibit autophagy [190]. Autophagy deficiency in keratinocytes not only reduced their function but also impaired their proliferation and migration. Therefore, the essential role of autophagy in the proliferation and migration of cells during wound healing should be considered for the development of biomaterials in the future. However, it is noteworthy that autophagic effects produced by various biomaterials may be different.

The formation of new vasculature through angiogenesis is considered an aspect of cutaneous wound regeneration that is critical for adequate healing [197]. Wound healing requires the formation of new capillaries for carrying nutrients, immune cells, and oxygen [198]. During adult wound healing, rapid and robust capillary growth creates a vascular bed, and as newly formed capillaries shrink with time, the density of the vascular system approaches that of normal skin. Under adverse conditions such as hypoxia or ischemia, autophagy maintains cell viability by recycling nutrients and generating energy. Several recent studies have associated intact autophagic responses with the homeostasis and function of ECs [199]. Furthermore, ECs suffer less oxidative damage when autophagy is active. Another study showed that curcumin induces autophagy in HUVECs treated with hydrogen peroxide (H_2_O_2_) by increasing the expression of LC3II and the number of autophagosomes to promote cell viability [200]. Biomaterials have also been shown to promote wound healing by enhancing angiogenesis via the modulation of autophagy. An et al. reported that MSCs can improve full-thickness cutaneous wound healing and tissue regeneration [147]. Mechanistically, MSCs can directly activate autophagy to promote ERK phosphorylation and VEGF paracrine secretion by accelerating the angiogenic capability of ECs (see Fig. 6d-f in Ref. [147]). In addition to controlling the survival or death of ECs, autophagy regulates other important functions, such as oxidative stress injury.

ROS are small, highly reactive and short-lived molecules that result from incomplete one-electron reduction of oxygen [201]. There are several types of ROS, including oxygen anions, free radicals, hydroxyl radicals and peroxides such as H_2_O_2_ [202]. Wound healing is greatly influenced by oxidative stress. The imbalance of free radicals and antioxidants in the body leads to excessive production of ROS, which eventually leads to tissue damage, cell death and delayed wound healing [203]. Therefore, decreased production of ROS may assist in reducing oxidative stress-induced damage and improving healing. This phenomenon was validated in our previous study, in which redox-sensitive poly(N-isopropylacrylamide-acrylic acid) nanogel-doped nanofibrous membranes promoted wound healing by adjusting ROS levels in wounded skin by mediating the redox potential [204]. In addition, a recent study showed that Fe-MIL-101_NH2 (a classic MOF) can maintain ROS levels and induce cytoprotective autophagy, instead of cytotoxicity, in mouse embryonic fibroblast cells [146]. Further investigation of the underlying mechanisms revealed that Fe-MIL-101_NH2 induced autophagy primarily by inhibiting the mTOR pathway and activating Beclin-1. In addition, elevated levels of autophagy were associated with an increase in ATG5 expression, which is essential for the maturation/termination of autophagy. Zhang et al. reported that nanofiber films synthesized using chitosan, polyvinylpyrrolidone and DHQ (CPD) had antioxidant activity, were nontoxic to human skin keratinocytes and increased the expression of pankeratin, VEGF and CD31 to promote wound healing by inducing autophagy (see Fig. 6g-i in Ref. [28] ).

In addition to the abovementioned mechanisms underlying skin wound healing, other mechanisms involve antimicrobial activity and anti-inflammatory effects. Therefore, advanced multifunctional materials with these properties should be developed in the future to promote skin regeneration.

**Fig. 6:**
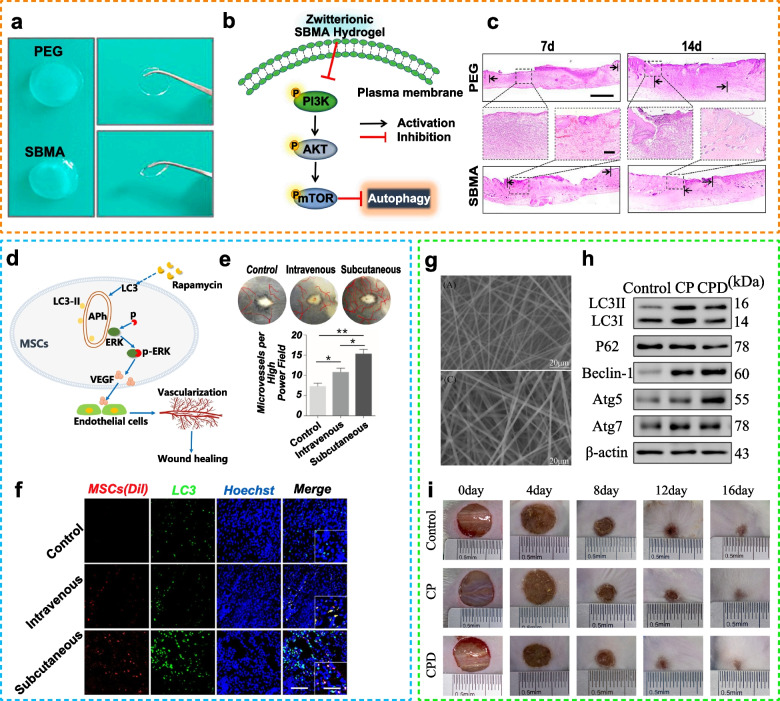
Autophagy-modulating biomaterials promote skin regeneration. **a** Comparison of the appearance of PEG and SBMA hydrogels. **b** Zwitterionic SBMA hydrogels inhibit the PI3K/Akt and mTOR signaling pathways to enhance autophagy. **c** Images of skin wounds stained with H&E on days 7 and 14 of treatment with PEG or SBMA hydrogels. (Reproduced with permission [118]; Copyright 2021, Frontiers Media S.A.) (**d**) Autophagy regulates ERK phosphorylation, which increases the release of VEGF from MSCs, and VEGF further promotes the vascularization of endothelial cells. **e** Assessment of capillary number in the wounded skin after 2 weeks of subcutaneous and intravenous injections of MSCs. **f** MSCs (red) and LC3-positive cells (green) 24 h after infusion (Reproduced with permission [147]; Copyright 2018, Nature Portfolio). **g** SEM images and diameter distribution. **h** Representative bands for LC3II/I, P62, Beclin-1, ATG5, and ATG7. **i** Representative images of wound healing on days 0, 4, 8, 12, and 16 of treatment with the nanocomposite films CP and CPD (Reproduced with permission [28]; Copyright 2022, Elsevier)

### Autophagy-based biomaterials for nerve regeneration

**Fig. 7:**
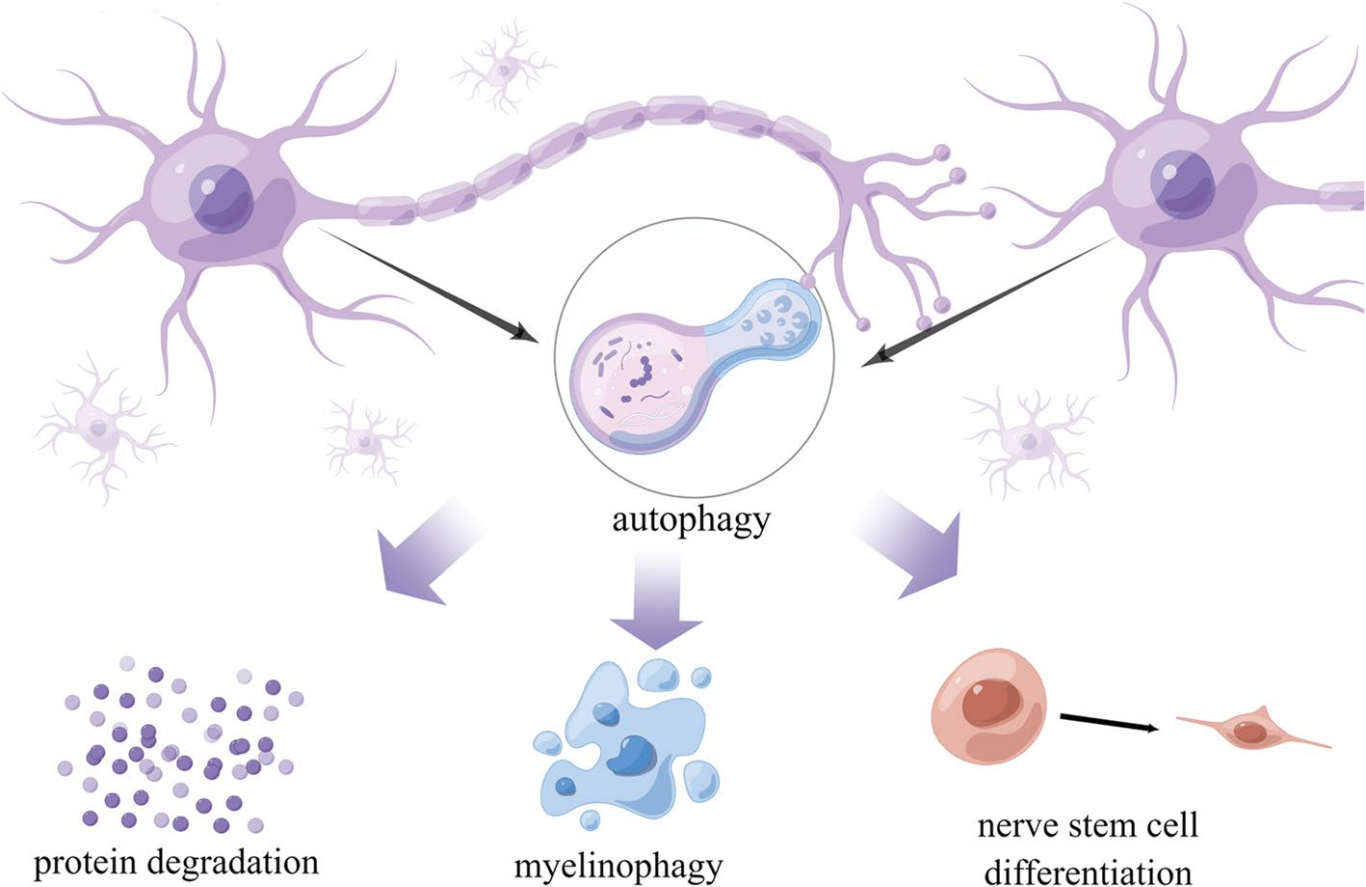
Schematic illustration of the mechanisms through which autophagy regulates nerve regeneration (drawn using Figdraw). Autophagy promotes nerve regeneration by clearing protein aggregates, promoting myelinophagy, and inducing the differentiation of neural stem cells. (Created with figdraw.com)

The nervous system regulates the activity of various organ systems and helps the human body elicit responses to multiple environmental changes. The metabolically active nervous system relies on catabolic processes to eliminate waste, provide energy, promote cell remodeling, and protect against pathogen invasion [205]. Nervous system disorders result in life-threatening consequences owing to the low ability of patients to rehabilitate damage. Several studies have focused on developing new strategies, including modulation of autophagy, for neural regeneration and rehabilitation. The mammalian nervous system requires autophagy to maintain normal function and homeostasis by protecting cells from various types of damage, including the aggregation of misfolded proteins and impaired organelles [205]. Additionally, autophagy influences the pathogenesis of nervous system disorders, including neurotrauma and neurodegeneration [206, 207]. Upregulation of autophagy exerts beneficial effects on neurodegeneration by increasing the degradation of abnormally accumulated proteins [206]. In addition to protecting neurons from neurodegeneration, autophagy promotes the differentiation of adult neural stem cells. However, impaired autophagy has been shown to result in premature death in ATG5-knockout (KO) mice with congenital malformation of the nervous system [208]. After nerve injury, selective autophagy in Schwann cells (SCs), called myelinophagy, removes myelin debris and accelerates nerve regeneration [209]. Therefore, regulation of autophagy represents a new strategy for treating nervous system disorders (Fig. 7).

Biomaterials can be used in the treatment of nervous system disorders as tissue-engineered scaffolds, drug delivery vehicles, and structural implants [210, 211]. Qian et al. reviewed the use of nanoparticles in peripheral nerve regeneration, which was primarily divided into three facets: scaffold construction, drug delivery, and microenvironment remodeling [212]. Cell-based biomaterials have been widely used to reconstruct damaged circuitry for the treatment of central neural diseases [210]. The utilization of biomaterials, either encapsulated with autophagy inducers or in their naked form, holds potential for the modulation of autophagy as a therapeutic approach for nervous system diseases. This implies that the regulation of autophagy should be taken into account during the design of biomaterials, emphasizing its significance in this context. In this review, we summarized recent advances in the use of biomaterials in promoting nerve regeneration and highlighted their potential role in autophagy regulation. Because most studies on autophagy-modulating biomaterials have focused on neurodegenerative diseases, we emphasized the potential interaction between biomaterials and autophagy in neurodegenerative diseases.

The accumulation of misfolded proteins is the major pathological phenotype of most late-onset neurodegenerative diseases [206]. Autophagy-induced clearance of abnormally accumulated proteins is a promising strategy for removing aggregated proteins and alleviating the symptoms of neurodegeneration [206]. AD is characterized by the accumulation of Aβ protein and phosphorylation of tau protein, leading to the loss of cellular homeostasis and, eventually, cell death. In a previous study, we demonstrated that ceramide can induce autophagy in cells. We developed PEG-ceramide nanomicelles and treated N2a cells with these nanomicelles to investigate their effects on autophagy and Aβ protein. PEGylated nanomicelles were found to increase autophagic flux and accelerate the degradation of Aβ protein in N2a cells. Therefore, a promising approach to developing nanomedicines against AD is acceleration of Aβ protein clearance, which is promoted through modulation of autophagy [213]. Furthermore, in mouse models of AD, functionalized single-walled carbon nanotubes can reverse abnormal mTOR signaling and alleviate impaired lysosomal proteolysis, thereby promoting the degradation of Aβ protein aggregates (see Fig. 8a-c in Ref. [90]). In addition to regulating the signaling pathway of autophagy, biomaterials can directly interact with Aβ protein aggregates. Luo et al. designed nanosweepers decorated with PEG-KLVFFF and Beclin-1 peptide (TGFQGSHWIHFTANFVNT), which can capture Aβ protein and upregulate autophagy to digest the protein [149]. In Parkinson’s disease (PD), α-synuclein (α-syn) accumulates in Lewy bodies along with selective denaturation and death of dopaminergic neurons in the substantia nigra [214]. In a study, curcumin analog-based nanoscavengers (NanoCA) were designed to induce overexpression of transcription factor EB (TFEB), an autophagy regulator, and promote autophagy-mediated degradation of α-syn. Exposure to NanoCA enhanced the calcium-dependent secretion of exosomes encapsulated with α-syn from PC12 cells. An intranasal drug delivery system was used for the targeted delivery of NanoCA to the mouse brain. This resulted in an improvement in the specificity of the nanomaterial to the central nervous system (see Fig. 8d-f in Ref. [150]). Aggregation of huntingtin (Htt) is a neuropathological hallmark of Huntington’s disease (HD). Wei et al. synthesized europium hydroxide [EuIII(OH)_3_] nanorods to promote autophagy in various cell lines, including Neuro 2a and PC12 cells, with GFP-Htt (with 74 polyQ repeats) aggregation. Treatment of these cells with EuIII(OH)_3_ nanorods enhanced the clearance of GFP-Htt (see Fig. 8g-j in Ref. [154]). Therefore, biomaterial-based autophagy exerts therapeutic effects by clearing protein aggregates through modulation of autophagy (mTOR), thus promoting the transcription of TFEB or directly targeting aggregates through surface modification.

Lysosomal function plays a significant role in reversing nerve degeneration. Lysosomes are a critical component of ALP, which is responsible for the degradation of intracellular materials, including protein aggregates, in neurodegenerative diseases. ALP relies on the critical acidic lysosomal pH (4.5–5.5) to optimize the function of hydrolases and ensure the fusion of lysosomes with autophagosomes [215]. Dysfunction of lysosomes is associated with the abnormal aggregation of proteins in neurodegeneration, especially PD [206]. Biomaterials can restore lysosomal function by modulating lysosomal pH, which increases autophagic flux. Bourdenx et al. demonstrated that PLGA nanoparticles (PLGA-NPs) restored impaired lysosomal function through reacidification of lysosomes and attenuated dopaminergic neurodegeneration in mice [216].

Nerve injury significantly affects the health of patients; however, currently available treatment strategies are unsatisfactory. After nerve injury, glial cells and SCs transform into regenerative phenotypes, thereby providing strong clues to triggering neuronal regeneration and resulting in the formation of the basal lamina. Simultaneously, SCs expressing stimulating factors create a favorable environment for nerve regeneration [217]. Wallerian degeneration in the distal stump of an injured nerve involves the physical fragmentation of axons, degeneration of axons and myelin, migration of macrophages, and proliferation of SCs [218]. Novel strategies for nerve regeneration include the use of nerve guidance scaffolds, pharmacotherapy, and cell-based therapy [219]. However, only a few studies have reported the use of autophagy-modulating biomaterials for treating peripheral nerve injury. In a study, a melatonin/polycaprolactone neural guide conduit was synthesized to induce autophagy in a rat model of 15-mm sciatic nerve defects. The conduit enhanced debris clearance and nerve proliferation, possibly by influencing energy metabolism, and hence restored the microenvironment of the injured nerve [148]. Jiang et al. used exosomes derived from microRNA (miR)-30d-5p-overexpressing adipose-derived stem cells (ADSCs) to reduce ischemic nerve injury. In vitro, the inflammatory response of primary microglia induced by oxygen and glucose deprivation (OGD) was reversed by exosomes enriched with miR-30d-5p. This anti-inflammatory effect was due to the inhibition of M1 microglial/macrophage polarization and the promotion of M2 microglial/macrophage polarization by miR-30d-5p. Furthermore, in a murine model of middle cerebral artery occlusion (MCAO), miR-30d-5p-enriched exosomes delivered via tail vein injection reduced the expression of the autophagy-related proteins Beclin-1, Atg5, and LC3 in brain tissue and decreased the cerebral injury area caused by infarction. These results indicated that exosomes overexpressing miR-30d-5p can promote the M2-type transformation of microglia by inhibiting autophagy, thereby reversing ischemic nerve injury [220]. Although both the promotion and inhibition of autophagy have therapeutic effects on nerve injury, the role of biomaterial-regulated autophagy in nerve injury remains unclear and warrants further investigation.

Biomaterials can be used as autophagy modulators in the treatment of nervous system disorders. In peripheral nervous system injury, biomaterial-based autophagy promotes nerve regeneration by providing energy and clearing debris. In central nervous system (CNS) injury, especially nerve degeneration, biomaterial-based autophagy plays an important role in clearing aggregated proteins and restoring lysosomal function. However, biomaterial-induced upregulation of autophagy also induces toxicity [221]. Moreover, the specific mechanism through which biomaterials promote autophagy in neurocytes remains unclear. Therefore, more studies should be conducted before such therapeutic approaches enter clinical trials.

**Fig. 8:**
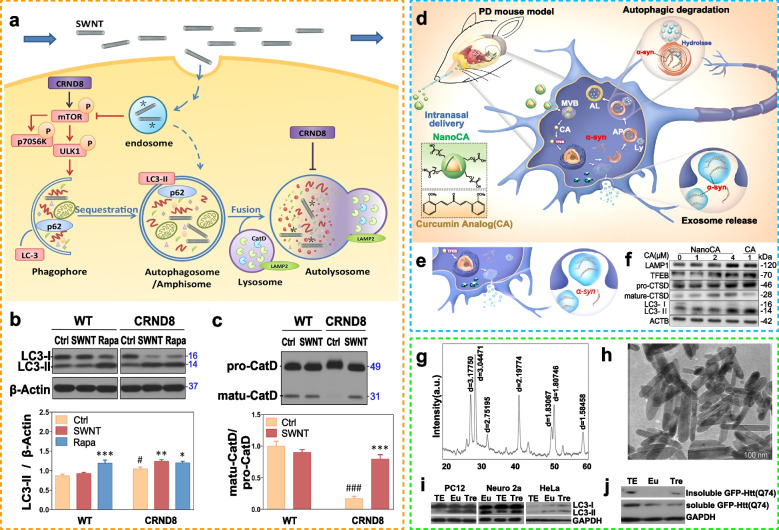
Autophagy-modulating biomaterials promote nerve regeneration. **a** Graphic illustration of the autophagy pathway and its impairment in CRND8 glial cells at two levels. **b** Western blotting for the quantification of LC3I and LC3II. **c** Immunoblots of pro-CatD and matu-CatD and their quantification (Reproduced with permission [90]; Copyright 2014, American Chemical Society). **d** Graphic illustration of NanoCA for TFEB-regulated cellular clearance of α-syn in experimental models of PD. **e** Schematic illustration of the effects of NanoCA on TFEB-mediated exosome release for α-syn clearance. **f** Western blotting of LC3II, TFEB, and LAMP1 in Neuro-2a cells after treatment with NanoCA (Reproduced with permission [150]; Copyright 2020, American Chemical Society). **g** XRD pattern of EuIII(OH)_3_ nanorods. **h** TEM image of EuIII(OH)_3_ nanorods. **i** Western blotting of soluble and insoluble GFP-Htt. **j** Western blotting of LC3I and LC3II in three types of cells treated with EuIII(OH)_3_ nanorods (Reproduced with permission [154]; Copyright 2014, Elsevier)

### Autophagy-based biomaterials for heart regeneration

The heart works as a pump with high energy consumption and blood supply. Owing to the limited regenerative capacity of adult cardiomyocytes, impairment of myocardiocytes results in myocardial remodeling, leading to interstitial fibrosis and, eventually, heart dysfunction [222]. Owing to the increasing incidence of heart failure and myocardial diseases, myocardial regeneration has become a major focus of studies attempting to elucidate the interplay between myocardial damage and regeneration. Autophagy plays a crucial role in regulating cardiac homeostasis and function by maintaining the basic structure and function of the heart through clearance of impaired organelles and misfolded proteins, which are activated during oxidative, metabolic, and genotoxic stress [223]. In a study, deficiency of ATG5 in mice resulted in failure to activate autophagy, and the mice rapidly died 12 h after birth owing to starvation, which manifested as hypoglycemia and myocardial damage. Therefore, autophagy is an important adaptive mechanism during neonatal starvation [224]. Autophagy plays an adaptive role in providing energy for myocardiocytes during ischemia. During acute infarction, autophagy is rapidly induced in myocardiocytes, and treatment with the autophagy inhibitor bafilomycin A1 increases the infarct size. Moreover, starvation and bafilomycin A1 neutralize their effects on infarct size, indicating that autophagy can protect the heart under energy-deprived conditions [225]. Autophagy can eliminate dysfunctional mitochondria, thereby promoting mitochondrial genesis and reducing the production of mitochondria-derived danger-associated molecular patterns (DAMPs). Overexpression of Beclin-1 can protect mitochondria by increasing mitophagy (degradation of dysfunctional mitochondria) through the PINK1-Parkin pathway, thereby attenuating cardiac damage in sepsis [226]. Therefore, autophagy plays an important role in reversing heart injury (see Fig. 9 in Ref. [227]).

**Fig. 9:**
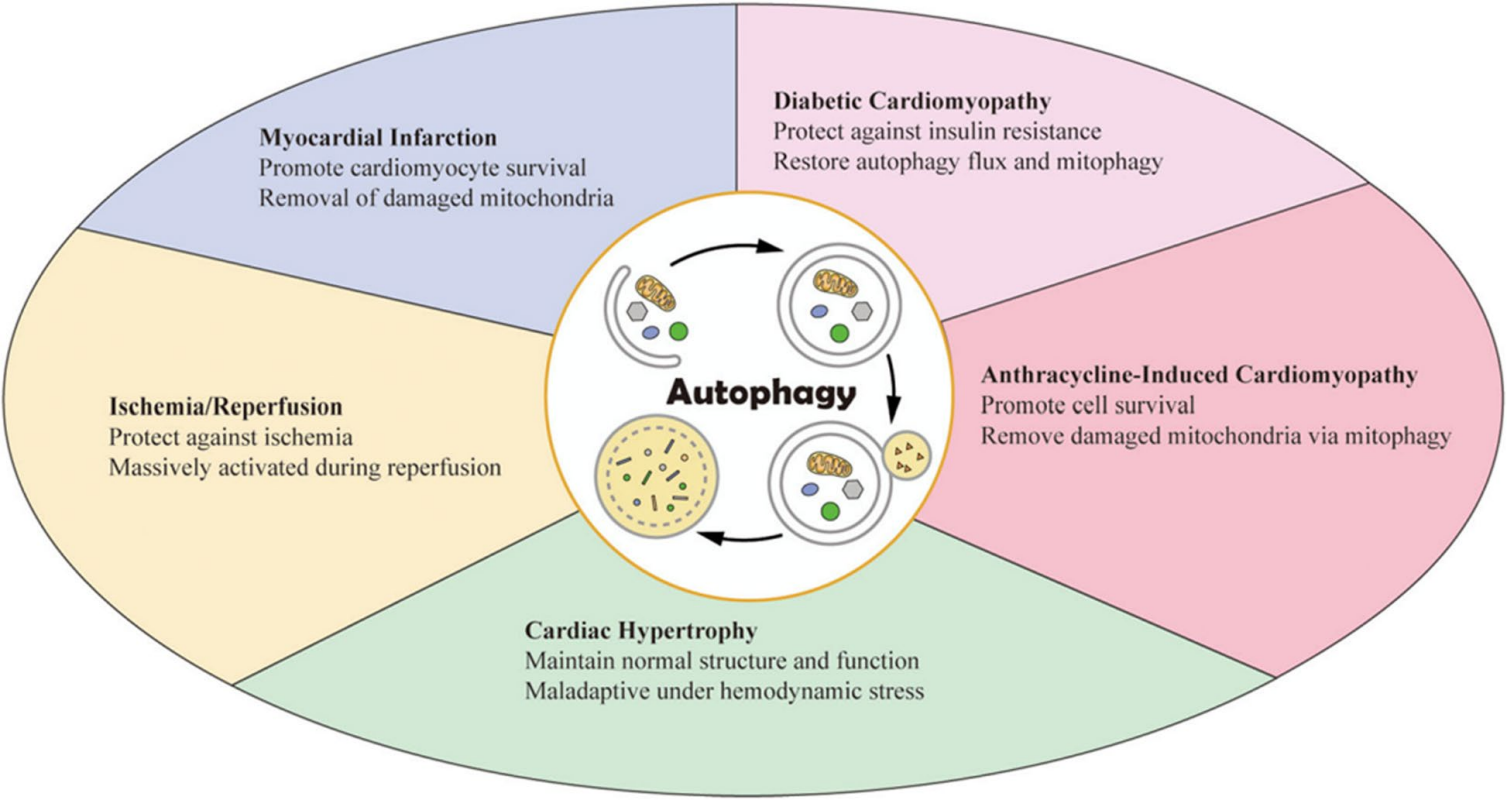
Role of autophagy in cardiac diseases. Autophagy facilitates regeneration in cardiac diseases and protects cardiomyocytes from injury. (Reproduced with permission [227]; Copyright 2021, John Wiley and Sons)

Biomaterials have been extensively used as vascular grafts, cardiac patches, and drug carriers in the treatment of cardiovascular diseases. Upregulation of autophagy by biomaterials encapsulated with autophagy inducers is beneficial for the treatment of heart diseases. The potential benefit offered by these agents lies in their ability to accumulate significantly at disease sites, while simultaneously allowing for lower serum levels of free drug, thereby minimizing the occurrence of off-target side effects. For example, biomaterials targeting the regulation of autophagy can be used to treat myocardial injury. Bibee et al. incorporated rapamycin in perfluorocarbon (PFC) NPs to formulate rapamycin-loaded nanoparticles (RNPs). The RNPs showed the ability to enhance autophagy in a mdx mouse model of Duchenne muscular dystrophy by inducing mTORC1 cell signaling and enhancing the phosphorylation of S6 ribosomal protein. These effects improved cardiac contractile performance, suggesting that the upregulation of autophagy plays a role in the treatment of congenital heart dystrophy (see Fig. 10a-c in Ref. [156]).

Oxidative stress is an important pathophysiological mechanism resulting in cardiac damage. Antioxidants can be combined with biomaterials to reduce toxicity and develop low-toxicity biomaterials. Shen et al. encapsulated N-acetylcysteine (NAC), a potent antioxidant, in magnetic mesoporous silica nanoparticles (M-MSNs). M-MSN@NAC inhibited the hyperactivation of autophagy and alleviated oxidative stress induced by exposure to iron oxide nanoparticles in cardiomyocytes (see Fig. 10d in Ref. [155]). The abovementioned studies suggest that both inducing and inhibiting excessive autophagy can facilitate cardiac regeneration.

In conclusion, autophagy is associated with oxidative stress and the metabolic state of the heart, especially after damage. Impaired autophagy contributes to insufficient energy supply and increases oxidative stress, which exacerbates myocardial injury. Modulating autophagy by restoring mitochondrial function and alleviating oxidative stress represents a novel strategy for treating heart injury. Given that biomaterials can improve drug delivery and can be internalized by cells, they can be combined with autophagy regulators to develop promising therapeutic approaches. However, the toxicity and biocompatibility of biomaterials should be considered before application. Moreover, the state of injury in which autophagy is activated and the beneficial effects of autophagy activation on the pathogenesis of myocardial injury remain unclear. To date, limited studies have focused on the use of autophagy-modulating biomaterials in the treatment of heart injury. Therefore, the effects of autophagy on heart injury and the underlying mechanisms governing these effects should be elucidated.

**Fig. 10:**
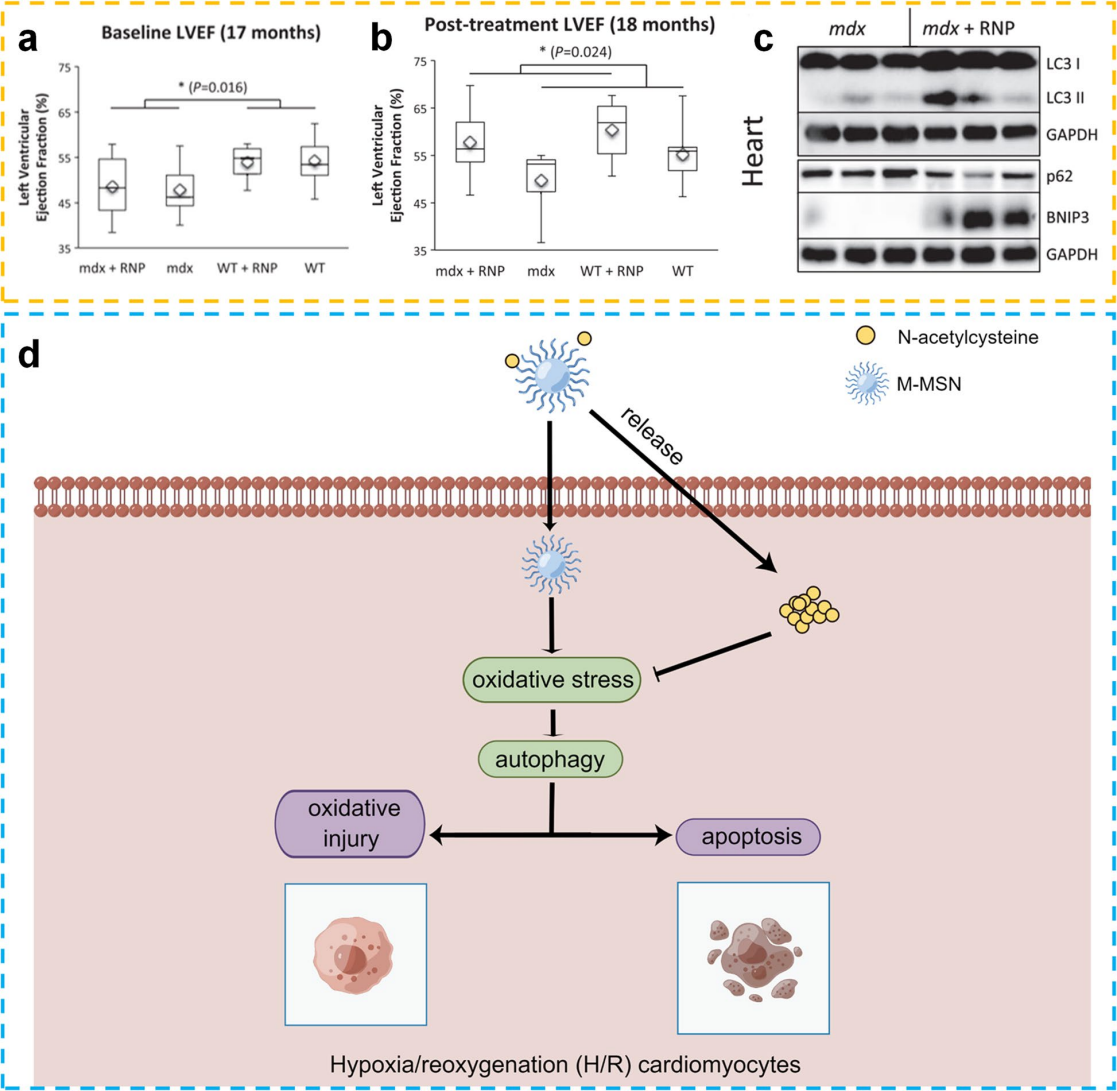
Autophagy-modulated biomaterials promote heart regeneration. **a** Baseline left ventricular ejection fraction of mdx and wild-type mice before treatment. **b** Improvement in cardiac function after treatment with rapamycin-loaded nanoparticles. **c** Western blotting of LC3I, LC3II, p62 and BNIP3 in the hearts of mdx mice treated with rapamycin-loaded nanoparticles. Reproduced with permission. [156] Copyright 2014, John Wiley and Sons. **d** Schematic illustration of the role of M-MSN@NAC in reducing oxidative injury and apoptosis by downregulating autophagy in cardiomyocytes stimulated with hypoxia and reoxygenation [155]

### Autophagy-based biomaterials for kidney regeneration

**Fig. 11:**
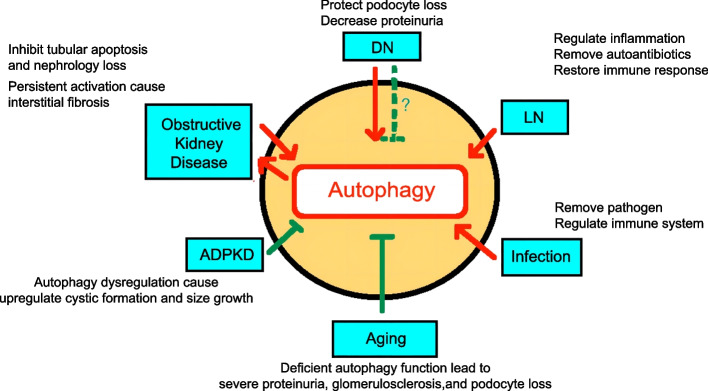
Illustration of the role of autophagy in chronic kidney disease. DN, diabetic nephropathy; LNm lupus nephropathy; APCKD, adult polycystic kidney disease. (Reproduced with permission [230]; Copyright 2019, MDPI)

The kidney is a complicated organ not only physiologically but also structurally and metabolically. After injury, the kidney undergoes multiple types of stress, such as hyperoxia, hypoxia, nutrient and energy depletion, ER stress, mitochondrial damage, and genotoxicity, all of which can activate autophagy. Figure 11 describes the pathophysiology of autophagy in chronic kidney disease. Studies have demonstrated the regulatory effects of autophagy on tissue homeostasis in the kidney and other organs using conditional gene-KO mice (tissue-specific ATG5- or ATG7-KO mice) and autophagy-deficient cells. Basal autophagy plays an important role in regulating cellular homeostasis in the kidney and other organs. Deficient autophagy can lead to protein aggregation and inclusion body accumulation in renal tubular cells [228]. Compared with wild-type mice, KO mice exhibit multiple signs of cellular senescence, including kidney function loss, mitochondrial damage, nuclear DNA damage, and fibrosis [228]. In addition, KO of the podocyte-specific ATG5 gene in mice leads to mild albuminuria, podocyte death, delayed glomerulonephritis, accumulation of oxidized and ubiquitinated protein aggregates, ER damage, and proteinuria [229]. Insufficient or defective autophagy results in impaired clearance of damaged macromolecules and organelles and cannot protect cells from damage in renal diseases. Biomaterials can be used to improve the therapeutic efficacy of currently available treatment strategies for kidney disease. Mechanistically, the anti-inflammatory, antioxidative, and antifibrotic properties of biomaterials may help to regulate autophagy.

Excessive formation of fibrous connective tissue in the kidney results in a progressive reduction in filtration efficiency and tubular function in both humans and experimental models of kidney disease [231]. In a previous review, we reported that fibrosis mainly occurs in two regions of the kidney: the tubulointerstitial space and glomerulus [105]. The final common outcome of many progressive kidney diseases is renal fibrosis, which may be an appropriate target for antifibrotic intervention in chronic kidney disease [105, 232]. Therapeutic strategies for reversing or inhibiting the progression of renal fibrosis are either inefficient or not safe enough to be translated into clinical practice [233]. Moreover, no drugs are available for treating kidney fibrosis. A recent study provided encouraging preclinical evidence indicating that the fibrogenesis pathway can be slowed, arrested, or reversed [234]. The kidney tubules rapidly undergo autophagy in response to side effects of drugs or acute pathological stimuli, such as ischemia and reperfusion injury [235, 236]. At the functional level, autophagy plays a largely protective role by preventing tubular damage and subsequent fibrosis in the kidney [237]. In a study, the relationship between autophagy and the treatment of kidney diseases was examined in a tubulointerstitial fibrosis (TIF) model constructed via intravenous injection of cationic bovine serum albumin (C-BSA) in Rab7 (a molecule involved in both endocytosis and autophagy)-overexpressing transgenic mice [157]. Albumin stimulation in the advanced stage of TIF triggered autophagy and MMP-2 activity, which are related to the progression of TIF, through upregulation of Rab7. Additionally, the use of Fe_3_O_4_ magnetic albumin NPs accelerated ECM degradation and decreased collagen accumulation through autophagy and inflammation (see Fig. 12a-c in Ref. [157]). An et al. designed phospholipid-polyethylene glycol-capped ceria-zirconia antioxidant nanoparticles (PEG-CZNPs), which reduced the expression of profibrotic cytokines in a human podocyte model of Fabry disease and attenuated kidney injury by enhancing autophagic flux [158].

Because inflammation leads to the occurrence of most kidney diseases, its regulation is key to the development of therapeutic strategies for kidney diseases. Many acute or chronic insults, such as ischemia, drug toxicity, accumulation of toxins, metabolism, and inflammation, can damage kidney tubules [238], resulting in the generation of inflammatory responses. The aging kidney also exhibits chronic inflammation of tubular lesions. Therefore, inhibition of renal inflammation is the primary therapeutic approach to reducing chronic renal injury and delaying the progression of kidney diseases [239]. NOD-like receptor family pyrin-containing domain-3 (NLRP3) is the most extensively investigated inflammasome that plays a vital role in numerous diseases [240]. After NLRP3 is activated in response to tissue damage, metabolic stress, and infection, it recruits apoptosis-associated speck-like protein with a caspase recruitment domain (ASC), leading to proteolytic cleavage of caspase-1. Cleavage of caspase-1 modulates the maturation of the proinflammatory cytokines IL-1β and IL-18, resulting in inflammation [241]. Lin et al. developed resveratrol-encapsulated PLGA-NPs, which not only improved the pharmacokinetic properties of resveratrol but also suppressed the NLRP3 inflammasome by enhancing autophagy in chronic kidney disease (see Fig. 12d in Ref. [27]). A further mechanism by which resveratrol NPs induce autophagy in kidney cells is by increasing AMPK and inhibiting pathways mediated by Akt and mTOR. These findings suggest that autophagy-induced anti-inflammatory activity can effectively treat chronic kidney disease. The novel nanoparticle-based drug delivery systems (NPs) exhibited precise control over drug release, while also offering the added advantage of convenient and direct targeting of drug delivery to injured kidney cells. Furthermore, these NPs demonstrated a reduction in negative side effects and yielded superior therapeutic effects compared to conventional drug delivery systems such as Res.

Under physiological conditions, ROS participate in the proliferation, differentiation, apoptosis, and immune defense of various cell types, including renal cells [242]. However, under pathological conditions, excessive ROS production causes irreversible damage to the structure and function of the kidney, eventually leading to end-stage renal disease. The redox regulation of proteins is mediated by ROS in several signaling pathways, including autophagy [243]. Autophagy plays a vital role in maintaining the homeostasis of podocytes, which are highly differentiated epithelial cells located on the surface of glomerular capillaries. Nano-TiO_2_ can trigger autophagy in kidney cells by inducing autophagic flux and activating AMPK to inhibit mTOR [75], and nano-TiO_2_-induced autophagy can act as an antioxidant system to prevent podocyte death (see Fig. 12e in Ref. [75]).

In conclusion, biomaterial-based autophagy plays a crucial role in the prevention of kidney diseases. Selective stress stimuli, such as ischemia-reperfusion injury, accumulation of toxins, and sepsis, can lead to acute damage, accumulation of ROS, and activation of autophagy in the normal kidney. Biomaterial-based autophagy can promote kidney regeneration by playing a protective role in podocytes, mesangial cells, and tubular cells.

**Fig. 12:**
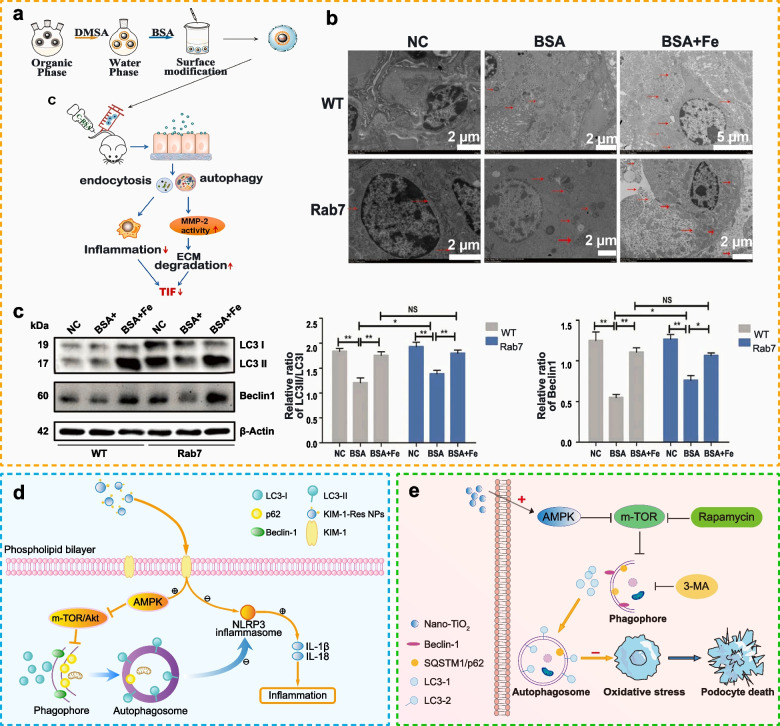
Autophagy-modulating biomaterials promote kidney regeneration. **a** Preparation of magnetic Fe_3_O_4_ nanoparticles encapsulated with albumin (Fe_3_O_4_@BSA) and illustration of the protective effects of Fe_3_O_4_@BSA on albumin-induced tubulointerstitial fibrosis. **b** Imaging of the structure of the kidney and autophagic vacuoles in the kidneys of mice after treatment. **c** Western blotting for the quantification of LC3II/LC3I levels in the kidneys of mice (Reproduced with permission [157]; Copyright 2021, Elsevier). **d** Schematic illustration of the role of KIM-1-Res NPs in ameliorating chronic kidney disease [27]. **e** Schematic illustration of the role of Nano-TiO_2_ in inducing autophagy to protect against cell death [75]

### Autophagy-based biomaterials for lung regeneration

The lungs exchange gases between the body and external environment through respiration, which is important for maintaining life. Various specialized cells work in coordination for proper functioning of the lungs, including pulmonary epithelial cells, interstitial fibroblasts, endotheliocytes, smooth muscle cells, and adventitial fibroblasts [244]. Lung components are directly exposed to the environment and provide the first line of defense against damage caused by chronic exposure to environmental (bacterial, viral, and oxidative) and mechanical stress [245]. Lung function is maintained by mechanisms such as antioxidant defense, innate and adaptive immune responses, and several cell death mechanisms. Autophagy, which is activated by both environmental and cellular stress stimuli, is crucial for maintaining cellular homeostasis (see Fig. 13 in Ref. [246]). The activity of autophagy is relatively low in most tissues but is greatly enhanced under stressful conditions. Disruption of cellular processes can lead to pathological conditions in the respiratory system. Autophagy plays an important role in maintaining lung homeostasis and preventing diseases such as chronic obstructive pulmonary disease (COPD), pulmonary vascular disease, asthma, and pulmonary fibrosis [247]. Biomaterial-based autophagy can regulate lung regeneration mainly by reducing the inflammatory response and inhibiting the proliferation of lung cells.

**Fig. 13:**
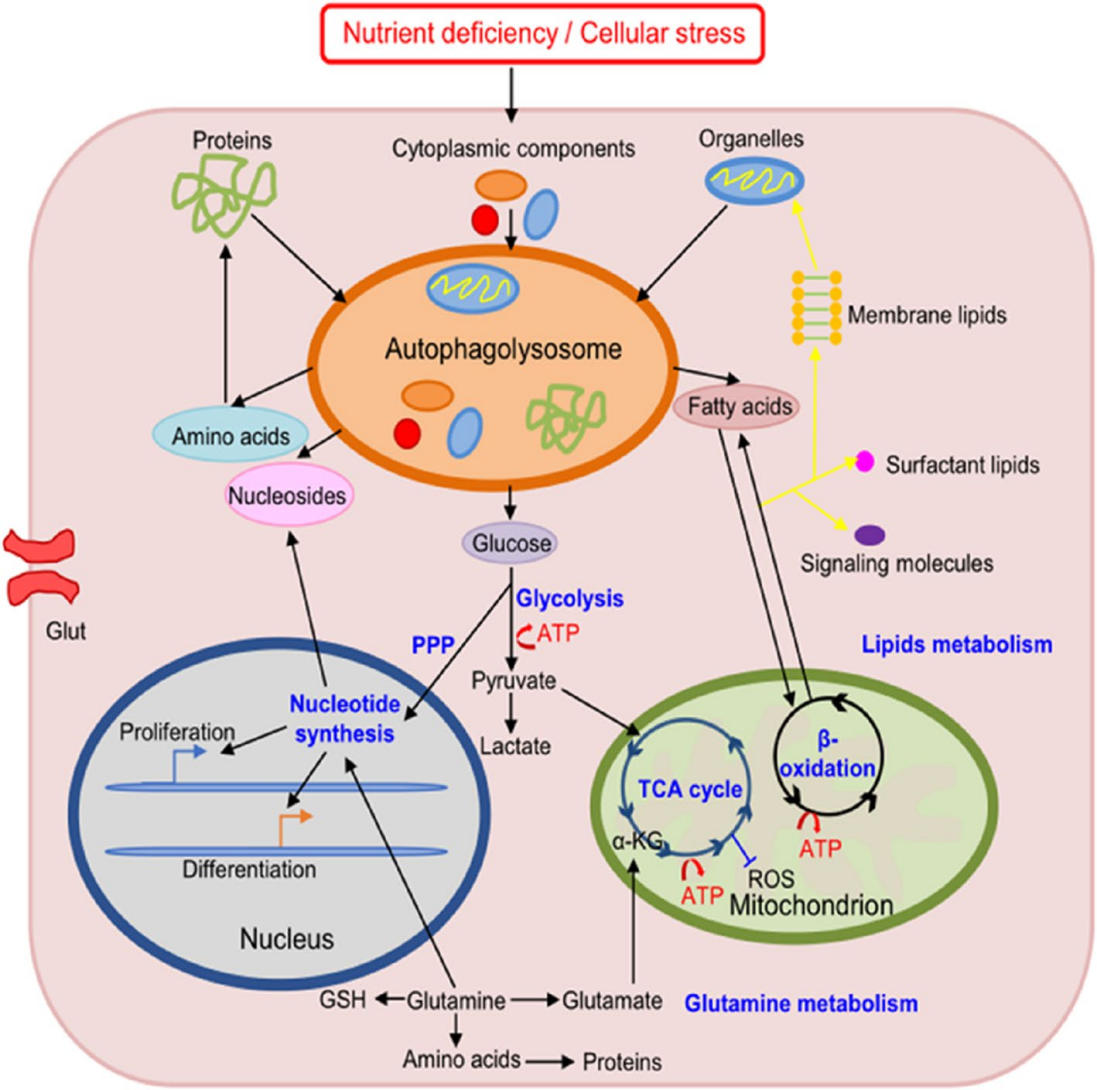
Underlying mechanism of autophagy in lung tissue. Nutrient deprivation and stress can trigger autophagy. ATP, adenosine triphosphate; α-KG, alpha-ketoglutarate; PPP, pentose-phosphate pathway; ROS, reactive oxygen species; TCA, tricarboxylic acid cycle. (Reproduced with permission [246]; Copyright 2021, Springer Nature)

Macrophages are one of the most important effector cells in the innate immune system of the lung, and their polarization plays a major role in the occurrence, development, and outcome of lung diseases [248]. In addition to generating the classical inflammatory response, macrophages infiltrated by invading entities exhibit a polarized phenotype, which is essential for maintaining the physiological functions of the host [249]. Lung macrophages can usually be classified into two subtypes based on their polarization: traditionally (M1) and alternatively (M2) activated macrophages [250]. Acute exudative lung inflammation activates M1 macrophages, which release abundant proinflammatory mediators and chemokines that recruit neutrophils and monocytes to the site of inflammation. In the late stages of tissue regeneration, lung macrophages polarize to the M2 phenotype to inhibit inflammation [251]. Autophagy plays a crucial role in regulating the function of macrophages. Huang et al. constructed a dual-therapeutic nanoplatform incorporating mTOR siRNA and spermidine (TETSpd-mTOR) to combat inflammation in acute lung injury (ALI) based on the properties of lung macrophages (see Fig. 14a in Ref. [17]). Spermidine neutralized DNA charges and facilitated the assembly of DNA tetrahedrons, which served as both initiators of the synthesis of DNA tetrahedron nanostructures and drugs in NPs. Spermidine serves not only as a mediator of drug delivery vehicle synthesis in TETSpd-mTOR but also as a drug that promotes macrophage autophagy via the AMPK-mTOR pathway. Notably, mTOR siRNA also reinforced macrophage autophagy. Therefore, spermidine and mTOR siRNA exerted synergistic effects on macrophage polarization and reduced inflammation, thus offering a novel therapeutic strategy for ALI.

Pulmonary arterial hypertension (PAH) is a complication of chronic lung disease in which blood pressure is increased in the small vessels of the lungs. It can cause pulmonary vascular resistance (PVR) and right ventricular hypertrophy, eventually resulting in right heart failure. During PAH, the pulmonary vascular system multiplies and remodels owing to alveolar hypoxia. The proliferation of pulmonary arterial smooth muscle cells (PASMCs) and their resistance to apoptosis play a key role in the development of PAH [252]. Previous studies have demonstrated that autophagy plays an essential role in pulmonary vascular diseases and exerts both protective and detrimental effects [253]. You et al. designed and developed a self-assembling DNA nanotube system [159] by incorporating mTOR siRNA as a functional agent into an overhang on a DNA nanotube through hybridization at a specific molar ratio. The self-assembled siRNA-DNA-NTs targeting mTOR exhibit notable stability, inherent biocompatibility, and minimal cytotoxicity. And these mTOR siRNA-loaded DNA nanotubes effectively facilitate the delivery of siRNA to pulmonary arterial smooth muscle cells (PASMCs), effectively suppressing their proliferation through the induction of autophagy, irrespective of normal or hypoxic conditions (see Fig. 14b-c in Ref [159]). However, the growth of pulmonary cells can be inhibited by reducing autophagy. PAH is an important pathophysiological condition that is characterized by persistent PVR and vasoconstriction in COPD and other clinical lung disorders [254]. Hypoxia, oxidative stress, starvation, and other aberrant conditions can impair the function of pulmonary ECs. During the progression of lung disease, aberrant proliferation of ECs is activated, and apoptosis is suppressed [255]. Du et al. synthesized triangular DNA nanoparticles encapsulated with ATG101 single-stranded antisense RNA (ssATG101-TNPs) and effectively transfected these NPs into human pulmonary arterial endothelial cells (PAECs) [136]. ssATG101-TNPs promoted apoptosis and inhibited the proliferation of PAECs by inhibiting macroautophagy under hypoxic conditions in a time- and dose-dependent manner.

Autophagy exerts either protective or harmful effects during the progression of lung disease. These diverse effects differ in different types of diseases and among lung cell types. However, the effects of autophagy on lung regeneration and metabolism remain elusive. Owing to the dynamic nature of autophagy, it is necessary to understand changes in its activity at varying stages of lung disease development.

**Fig. 14:**
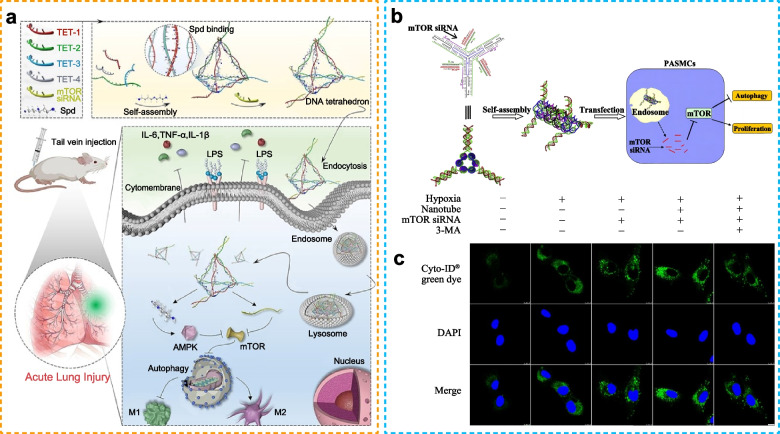
Autophagy-modulating biomaterials promote lung regeneration. **a** Schematic illustration of the synergistic anti-inflammatory effects of TETSpd-mTOR on ALI (Reproduced with permission [17]; Copyright 2022, John Wiley and Sons). **b** Schematic illustration demonstrating self-assembled mTOR siRNA-loaded nanotubes that stimulate PASMC proliferation and autophagy. **c** Fluorescence imaging demonstrating autophagy in pulmonary arterial smooth muscle cells incubated with mTOR siRNA under hypoxic conditions (Reproduced with permission [159]; Copyright 2015, Elsevier)

### Autophagy-based biomaterials for liver regeneration

The liver is one of the most dynamic metabolic organs that is essential for the synthesis of plasma proteins, glucose, glycogen, cholesterol, lipids, and fatty acids and the metabolism and detoxification of drugs/xenobiotics [256]. Autophagy is important for maintaining the balance between liver metabolism and cellular health [257]. Physiologically, autophagy plays an essential role in preserving cellular and metabolic homeostasis in the liver. Nervous, endocrine, and paracrine signals coordinated with circulating nutrients are responsible for orchestrating liver autophagy [256]. Dysfunction of autophagy is associated with several liver diseases, including hepatitis, steatosis, fibrosis, cirrhosis, and hepatocellular carcinoma [257]. Autophagy protects liver cells from injury and death by removing damaged organelles and proteins. Modulating autophagy can alter the occurrence and outcome of liver diseases, suggesting that autophagy is a novel therapeutic target for preventing and treating liver diseases.

Biomaterials can reduce inflammation by regulating autophagy in liver injury-related diseases [161]. Sepsis has a high rate of morbidity and mortality among patients admitted to the intensive care unit worldwide, and an excessive inflammatory response leads to liver injury. Superparamagnetic iron oxide NPs (SPIONs) synthesized using γ-Fe_2_O_3_ NPs can alleviate LPS-induced sepsis and prevent the infiltration of inflammatory cells into the liver. Mechanistically, SPIONs directly stimulate macrophage autophagy by activating the Cav1-Notch1/HES1 signaling pathway, which enhances the production of IL-10 in macrophages and consequently inhibits inflammation in LPS-induced sepsis and liver injury.

## Conclusions and future perspectives

Autophagy, a lysosome-dependent clearance mechanism, maintains cellular homeostasis by mediating the degradation of senescent organelles, pathogens, and abnormal proteins and is closely associated with tissue regeneration. It can promote tissue regeneration through the following mechanisms: effective removal of intracellular bacteria to alleviate infection, negative regulation of inflammation, promotion of cell survival by inhibiting apoptosis, promotion of the differentiation and migration of cells, and formation of ECM. However, excessive autophagy can lead to overdegradation of cellular contents and trigger autophagic cell death, which may impede tissue regeneration. Several biomaterials can be used to induce autophagy for a wide range of applications in tissue regeneration. Autophagy induced by biomaterials in specific cells can promote tissue regeneration. An appropriate level of autophagy can enhance cell metabolism and is necessary for the cellular energy cycle to maintain homeostasis, whereas excessive autophagy depletes the cellular architecture and maintains the cell in a state of stress, eventually leading to programmed cell death. Therefore, elucidating the molecular mechanisms underlying autophagy induced by different biomaterials and regulating autophagy at the molecular level are important approaches to developing effective strategies for promoting tissue regeneration.

Surface modifications and alterations in the structure of biomaterials, especially multicomponent nanomaterials, can increase or decrease autophagic flux in tissue cells. In addition, controlling biomaterial-induced cytotoxicity is critical for changing the direction of autophagy induction. Specifically, materials and their size, shape, charges, and surface modifications should be optimized for the efficient and safe use of biomaterials. Biomaterials incorporating autophagy inducers also possess the ability to regulate autophagy by either activating or inhibiting its processes, thereby facilitating drug delivery. Moreover, understanding the mechanisms underlying nanomaterial-induced autophagy is critical for promoting tissue regeneration. Stress, such as ER stress and organelle damage, is the primary trigger of nanomaterial-induced autophagy. Additionally, nanomaterial-induced molecular perturbations and stimulation of related receptors can contribute to the activation of autophagy and immune regulation. Biomaterial-induced autophagy regulates the immune function of tissue cells through multiple mechanisms, including proliferation, migration, apoptosis, and differentiation, which are important for the survival and function of cells in the tissue microenvironment.

Autophagy maintains cellular homeostasis by eliminating damaged organelles and cell debris and is associated with the molecular pathways that control intracellular immune activity. Autophagy induced by biomaterials can protect cells from both extracellular and intracellular stress stimuli and support the proliferation, survival, and differentiation of tissue cells. Further research into the principle and pathways of autophagy regulation in tissue cells can promote the development and optimization of strategies for promoting tissue regeneration. Biomaterials with features of foreign bodies have emerged as important agents that can regulate autophagy by disrupting the normal physiological functions of tissue cells. Understanding opsonization and the molecular mechanisms underlying biomaterial-based autophagy can facilitate the rational design of biomaterials. Autophagy-modulating biomaterials play a positive role in promoting tissue repair and may be used to develop tissue regeneration approaches for clinical application.

For successful clinical use of biomaterial-based approaches, the dose and administration frequency of biomaterials should be optimized, and their clinical safety and performance should be improved. Additionally, the combination of biomaterials with other technologies and further expansion of the applications of biomaterials may help to maximize the therapeutic effects of tissue regeneration strategies.

## References

[1] Xie Z, Klionsky DJ (2007). Autophagosome formation: core machinery and adaptations. Nat Cell Biol.

[2] Klionsky DJ (2007). Autophagy: from phenomenology to molecular understanding in less than a decade. Nat Rev Mol Cell Biol.

[3] Antonioli M, Di Rienzo M, Piacentini M, Fimia GM (2017). Emerging mechanisms in initiating and terminating Autophagy. Trends Biochem Sci.

[4] Levine B, Kroemer G (2019). Biological Functions of Autophagy genes: a Disease Perspective. Cell.

[5] Li L, Tan J, Miao Y, Lei P, Zhang Q (2015). ROS and Autophagy: interactions and Molecular Regulatory Mechanisms. Cell Mol Neurobiol.

[6] Kalluri R, LeBleu VS (2020). The biology, function, and biomedical applications of exosomes. Science.

[7] Chen F-M, Zhang M, Wu Z-F (2010). Toward delivery of multiple growth factors in tissue engineering. Biomaterials.

[8] Sun M, Tan L, Hu M (2021). The role of autophagy in hepatic fibrosis. Am J Transl Res.

[9] Guan J-L, Simon AK, Prescott M, Menendez JA, Liu F, Wang F (2013). Autophagy in stem cells. Autophagy.

[10] Wang C, Telpoukhovskaia MA, Bahr BA, Chen X, Gan L (2018). Endo-lysosomal dysfunction: a converging mechanism in neurodegenerative diseases. Curr Opin Neurobiol.

[11] Paskeh MDA, Entezari M, Clark C, Zabolian A, Ranjbar E, Farahani MV (2022). Targeted regulation of autophagy using nanoparticles: New insight into cancer therapy. Biochim Biophys Acta Mol Basis Dis.

[12] Gomes LC, Dikic I (2014). Autophagy in antimicrobial immunity. Mol Cell.

[13] Loi M, Müller A, Steinbach K, Niven J, Barreira da Silva R, Paul P (2016). Macroautophagy proteins control MHC class I levels on dendritic cells and shape anti-viral CD8(+) T cell responses. Cell Rep.

[14] Prieto P, Rosales-Mendoza CE, Terrón V, Toledano V, Cuadrado A, López-Collazo E (2015). Activation of autophagy in macrophages by pro-resolving lipid mediators. Autophagy.

[15] Eming SA, Wynn TA, Martin P (2017). Inflammation and metabolism in tissue repair and regeneration. Science.

[16] Real E, Rodrigues L, Cabal GG, Enguita FJ, Mancio-Silva L, Mello-Vieira J (2018). Plasmodium UIS3 sequesters host LC3 to avoid elimination by autophagy in hepatocytes. Nat Microbiol.

[17] Huang C, You Q, Xu J, Wu D, Chen H, Guo Y (2022). An mTOR siRNA-Loaded Spermidine/DNA tetrahedron nanoplatform with a synergistic anti-inflammatory effect on Acute Lung Injury. Adv Healthc Mater.

[18] Li L, Yang S, Xu L, Li Y, Fu Y, Zhang H (2019). Nanotopography on titanium promotes osteogenesis via autophagy-mediated signaling between YAP and β-catenin. Acta Biomater.

[19] Mahapatra C, Lee R, Paul MK (2022). Emerging role and promise of nanomaterials in organoid research. Drug Discov Today.

[20] Park I-S, Mahapatra C, Park JS, Dashnyam K, Kim J-W, Ahn JC (2020). Revascularization and limb salvage following critical limb ischemia by nanoceria-induced Ref-1/APE1-dependent angiogenesis. Biomaterials.

[21] Mahapatra C, Singh RK, Lee J-H, Jung J, Hyun JK, Kim H-W (2017). Nano-shape varied cerium oxide nanomaterials rescue human dental stem cells from oxidative insult through intracellular or extracellular actions. Acta Biomater.

[22] Mahapatra C, Singh RK, Kim J-J, Patel KD, Perez RA, Jang J-H (2016). Osteopromoting Reservoir of Stem cells: bioactive mesoporous Nanocarrier/Collagen gel through slow-releasing FGF18 and the activated BMP signaling. ACS Appl Mater Interfaces.

[23] Mahapatra C, Kim J-J, Lee J-H, Jin G-Z, Knowles JC, Kim H-W (2019). Differential chondro- and osteo-stimulation in three-dimensional porous scaffolds with different topological surfaces provides a design strategy for biphasic osteochondral engineering. J Tissue Eng.

[24] Batool F, Özçelik H, Stutz C, Gegout P-Y, Benkirane-Jessel N, Petit C (2021). Modulation of immune-inflammatory responses through surface modifications of biomaterials to promote bone healing and regeneration. J Tissue Eng.

[25] Dikic I, Elazar Z (2018). Mechanism and medical implications of mammalian autophagy. Nat Rev Mol Cell Biol.

[26] Stern ST, Adiseshaiah PP, Crist RM (2012). Autophagy and lysosomal dysfunction as emerging mechanisms of nanomaterial toxicity. Part Fibre Toxicol.

[27] Lin Y-F, Lee Y-H, Hsu Y-H, Chen Y-J, Lin Y-F, Cheng F-Y (2017). Resveratrol-loaded nanoparticles conjugated with kidney injury molecule-1 as a drug delivery system for potential use in chronic kidney disease. Nanomed (Lond).

[28] Zhang J, Chen K, Ding C, Sun S, Zheng Y, Ding Q (2022). Fabrication of chitosan/PVP/dihydroquercetin nanocomposite film for in vitro and in vivo evaluation of wound healing. Int J Biol Macromol.

[29] Feng Y, He D, Yao Z, Klionsky DJ (2014). The machinery of macroautophagy. Cell Res.

[30] Li D, Wang C, Li Z, Wang H, He J, Zhu J (2018). Nano-sized Al2O3 particle-induced autophagy reduces osteolysis in aseptic loosening of total hip arthroplasty by negative feedback regulation of RANKL expression in fibroblasts. Cell Death Dis.

[31] Prasanna P, Rathee S, Upadhyay A, Sulakshana S (2021). Nanotherapeutics in the treatment of acute respiratory distress syndrome. Life Sci.

[32] Qiao Q, Liu X, Yang T, Cui K, Kong L, Yang C (2021). Nanomedicine for acute respiratory distress syndrome: the latest application, targeting strategy, and rational design. Acta Pharm Sin B.

[33] Gao J, Wang S, Wang Z (2017). High yield, scalable and remotely drug-loaded neutrophil-derived extracellular vesicles (EVs) for anti-inflammation therapy. Biomaterials.

[34] Xu K, An N, Zhang H, Zhang Q, Zhang K, Hu X (2020). Sustained-release of PDGF from PLGA microsphere embedded thermo-sensitive hydrogel promoting wound healing by inhibiting autophagy. J Drug Deliv Sci Technol.

[35] Kim KH, Lee M-S (2014). Autophagy–a key player in cellular and body metabolism. Nat Rev Endocrinol.

[36] Mahapatra KK, Panigrahi DP, Praharaj PP, Bhol CS, Patra S, Mishra SR (2019). Molecular interplay of autophagy and endocytosis in human health and diseases. Biol Rev Camb Philos Soc.

[37] Migneault F, Hébert M-J (2021). Autophagy, tissue repair, and fibrosis: a delicate balance. Matrix Biol.

[38] Wen X, Yang Y, Klionsky DJ (2021). Moments in autophagy and disease: past and present. Mol Aspects Med.

[39] Hansen M, Rubinsztein DC, Walker DW (2018). Autophagy as a promoter of longevity: insights from model organisms. Nat Rev Mol Cell Biol.

[40] White E, Mehnert JM, Chan CS (2015). Autophagy, metabolism, and Cancer. Clin Cancer Res.

[41] Ceccariglia S, Cargnoni A, Silini AR, Parolini O (2020). Autophagy: a potential key contributor to the therapeutic action of mesenchymal stem cells. Autophagy.

[42] Kim HD, Kong E, Kim Y, Chang J-S, Kim J (2017). RACK1 depletion in the ribosome induces selective translation for non-canonical autophagy. Cell Death Dis.

[43] Chu P, He L, Huang R, Liao L, Li Y, Zhu Z (2020). Autophagy inhibits Grass Carp Reovirus (GCRV) replication and protects Ctenopharyngodon idella kidney (CIK) cells from excessive inflammatory responses after GCRV infection. Biomolecules.

[44] Li X, He S, Ma B (2020). Autophagy and autophagy-related proteins in cancer. Mol Cancer.

[45] Su L-Y, Li H, Lv L, Feng Y-M, Li G-D, Luo R (2015). Melatonin attenuates MPTP-induced neurotoxicity via preventing CDK5-mediated autophagy and SNCA/α-synuclein aggregation. Autophagy.

[46] Levine B, Kroemer G (2008). Autophagy in the pathogenesis of disease. Cell.

[47] Li X, Kim J, Wu J, Ahamed AI, Wang Y, Martins-Green M (2020). N-Acetyl-cysteine and mechanisms involved in Resolution of Chronic Wound Biofilm. J Diabetes Res.

[48] Kim MJ, Nagy LE, Park P-H (2014). Globular adiponectin inhibits Ethanol-Induced reactive oxygen species production through modulation of NADPH oxidase in macrophages: involvement of liver kinase B1/AMP-Activated protein kinase pathway. Mol Pharmacol.

[49] Scherz-Shouval R, Elazar Z (2011). Regulation of autophagy by ROS: physiology and pathology. Trends Biochem Sci.

[50] Baechler BL, Bloemberg D, Quadrilatero J (2019). Mitophagy regulates mitochondrial network signaling, oxidative stress, and apoptosis during myoblast differentiation. Autophagy.

[51] Lee J, Giordano S, Zhang J (2012). Autophagy, mitochondria and oxidative stress: cross-talk and redox signalling. Biochem J.

[52] Levonen A-L, Hill BG, Kansanen E, Zhang J, Darley-Usmar VM (2014). Redox regulation of antioxidants, autophagy, and the response to stress: implications for electrophile therapeutics. Free Radic Biol Med.

[53] Cabello-Verrugio C, Ruiz-Ortega M, Mosqueira M, Simon F (2016). Oxidative stress in Disease and Aging: mechanisms and therapies. Oxid Med Cell Longev.

[54] Phumsuay R, Muangnoi C, Dasuni Wasana PW, Hasriadi H, Vajragupta O, Rojsitthisak P, et al. Molecular Insight into the Anti-Inflammatory Effects of the Curcumin Ester Prodrug Curcumin Diglutaric Acid In Vitro and In Vivo. IJMS. 2020;21:5700. 10.3390/ijms21165700 PMC7461142 32784830

[55] Deretic V, Levine B (2018). Autophagy balances inflammation in innate immunity. Autophagy.

[56] Kim J-W, Mahapatra C, Hong J-Y, Kim MS, Leong KW, Kim H-W (2017). Functional recovery of Contused spinal cord in rat with the injection of optimal-dosed Cerium Oxide Nanoparticles. Adv Sci (Weinh).

[57] Li C, Qu L, Farragher C, Vella A, Zhou B (2019). MicroRNA regulated macrophage activation in obesity. J Transl Int Med.

[58] Zhang S, Xie F, Li K, Zhang H, Yin Y, Yu Y (2022). Gold nanoparticle-directed autophagy intervention for antitumor immunotherapy via inhibiting tumor-associated macrophage M2 polarization. Acta Pharm Sin B.

[59] Chen W, Ma T, Shen X, Xia X, Xu G, Bai X (2012). Macrophage-induced tumor angiogenesis is regulated by the TSC2-mTOR pathway. Cancer Res.

[60] Roca H, Varsos ZS, Sud S, Craig MJ, Ying C, Pienta KJ (2009). CCL2 and interleukin-6 promote survival of human CD11b + peripheral blood mononuclear cells and induce M2-type macrophage polarization. J Biol Chem.

[61] Das LM, Binko AM, Traylor ZP, Peng H, Lu KQ (2019). Vitamin D improves sunburns by increasing autophagy in M2 macrophages. Autophagy.

[62] Xiao L, Xiao Y (2019). The Autophagy in Osteoimmonology: Self-Eating, maintenance, and Beyond. Front Endocrinol (Lausanne).

[63] El-Fiqi A, Mandakhbayar N, Jo SB, Knowles JC, Lee J-H, Kim H-W (2021). Nanotherapeutics for regeneration of degenerated tissue infected by bacteria through the multiple delivery of bioactive ions and growth factor with antibacterial/angiogenic and osteogenic/odontogenic capacity. Bioact Mater.

[64] Levine B, Mizushima N, Virgin HW (2011). Autophagy in immunity and inflammation. Nature.

[65] Lawrence DW, Kornbluth J (2012). E3 ubiquitin ligase NKLAM is a macrophage phagosome protein and plays a role in bacterial killing. Cell Immunol.

[66] Mizushima N, Yoshimori T, Ohsumi Y (2011). The role of atg proteins in autophagosome formation. Annu Rev Cell Dev Biol.

[67] Mathieu J (2015). Interactions between Autophagy and bacterial toxins: targets for Therapy?. Toxins (Basel)..

[68] Lodder J, Denaës T, Chobert M-N, Wan J, El-Benna J, Pawlotsky J-M (2015). Macrophage autophagy protects against liver fibrosis in mice. Autophagy.

[69] Chiu H-C, Soni S, Kulp SK, Curry H, Wang D, Gunn JS (2009). Eradication of intracellular Francisella tularensis in THP-1 human macrophages with a novel autophagy inducing agent. J Biomed Sci.

[70] Campbell GR, Spector SA (2012). Vitamin D inhibits human immunodeficiency virus type 1 and Mycobacterium tuberculosis infection in macrophages through the induction of autophagy. PLoS Pathog.

[71] Wu J, Duan R, Cao H, Field D, Newnham CM, Koehler DR (2008). Regulation of epithelium-specific ets-like factors ESE-1 and ESE-3 in airway epithelial cells: potential roles in airway inflammation. Cell Res.

[72] Singh I, Mehta A, Contreras A, Boettger T, Carraro G, Wheeler M (2014). Hmga2 is required for canonical WNT signaling during lung development. BMC Biol.

[73] Chen S, Wang W, Tan H-Y, Lu Y, Li Z, Qu Y (2021). Role of Autophagy in the maintenance of stemness in adult stem cells: a disease-relevant mechanism of action. Front Cell Dev Biol.

[74] Pan H, Cai N, Li M, Liu G-H, Izpisua Belmonte JC (2013). Autophagic control of cell stemness. EMBO Mol Med.

[75] Zhang X, Yin H, Li Z, Zhang T, Yang Z (2016). Nano-TiO2 induces autophagy to protect against cell death through antioxidative mechanism in podocytes. Cell Biol Toxicol.

[76] Mizushima N, Levine B, Cuervo AM, Klionsky DJ (2008). Autophagy fights disease through cellular self-digestion. Nature.

[77] Song S, Tan J, Miao Y, Li M, Zhang Q (2017). Crosstalk of autophagy and apoptosis: involvement of the dual role of autophagy under ER stress. J Cell Physiol.

[78] Shen J, Yang D, Zhou X, Wang Y, Tang S, Yin H (2019). Role of Autophagy in Zinc Oxide Nanoparticles-Induced apoptosis of mouse LEYDIG cells. IJMS.

[79] Shen S, Weng R, Li L, Xu X, Bai Y, Liu H (2016). Metabolomic analysis of mouse embryonic fibroblast cells in response to Autophagy Induced by Acute Starvation. Sci Rep.

[80] Ren H, Zhao F, Zhang Q, Huang X, Wang Z (2022). Autophagy and skin wound healing. Burns Trauma.

[81] Li C, Li J, Xu G, Sun H (2020). Influence of chronic ethanol consumption on apoptosis and Autophagy following transient focal cerebral ischemia in male mice. Sci Rep.

[82] Abdulghani S, Mitchell GR (2019). Biomaterials for in situ tissue regeneration: a review. Biomolecules.

[83] Li JJ, Hartono D, Ong C-N, Bay B-H, Yung L-YL (2010). Autophagy and oxidative stress associated with gold nanoparticles. Biomaterials.

[84] Xiao H, Yang X, Luo L-H, Ning Z (2018). Graphene oxide regulates endoplasmic reticulum stress: autophagic pathways in nasopharyngeal carcinoma cells. Int J Clin Exp Pathol.

[85] Lin T, Zhang Q, Yuan A, Wang B, Zhang F, Ding Y (2020). Synergy of Tumor Microenvironment Remodeling and Autophagy Inhibition to Sensitize Radiation for bladder Cancer Treatment. Theranostics.

[86] Wang Y, Lin Y-X, Qiao Z-Y, An H-W, Qiao S-L, Wang L (2015). Self-assembled autophagy-inducing polymeric nanoparticles for breast cancer interference in-vivo. Adv Mater.

[87] Mohammadinejad R, Moosavi MA, Tavakol S, Vardar DÖ, Hosseini A, Rahmati M (2019). Necrotic, apoptotic and autophagic cell fates triggered by nanoparticles. Autophagy.

[88] Ma X, Wu Y, Jin S, Tian Y, Zhang X, Zhao Y (2011). Gold nanoparticles induce autophagosome accumulation through size-dependent nanoparticle uptake and lysosome impairment. ACS Nano.

[89] Zhang Q, Yang W, Man N, Zheng F, Shen Y, Sun K (2009). Autophagy-mediated chemosensitization in cancer cells by fullerene C60 nanocrystal. Autophagy.

[90] Xue X, Wang L-R, Sato Y, Jiang Y, Berg M, Yang D-S (2014). Single-walled carbon nanotubes alleviate autophagic/lysosomal defects in primary glia from a mouse model of Alzheimer’s disease. Nano Lett.

[91] Jin P, Wei P, Zhang Y, Lin J, Sha R, Hu Y (2016). Autophagy-mediated clearance of ubiquitinated mutant huntingtin by graphene oxide. Nanoscale.

[92] Ji X, Xu B, Yao M, Mao Z, Zhang Y, Xu G (2016). Graphene oxide quantum dots disrupt autophagic flux by inhibiting lysosome activity in GC-2 and TM4 cell lines. Toxicology.

[93] Lin J, Huang Z, Wu H, Zhou W, Jin P, Wei P (2014). Inhibition of autophagy enhances the anticancer activity of silver nanoparticles. Autophagy.

[94] Xu Y, Wang L, Bai R, Zhang T, Chen C (2015). Silver nanoparticles impede phorbol myristate acetate-induced monocyte-macrophage differentiation and autophagy. Nanoscale.

[95] Li C, Liu H, Sun Y, Wang H, Guo F, Rao S (2009). PAMAM nanoparticles promote acute lung injury by inducing autophagic cell death through the Akt-TSC2-mTOR signaling pathway. J Mol Cell Biol.

[96] Wang J, Li Y, Duan J, Yang M, Yu Y, Feng L (2018). Silica nanoparticles induce autophagosome accumulation via activation of the EIF2AK3 and ATF6 UPR pathways in hepatocytes. Autophagy.

[97] Zhang J, Zou Z, Wang B, Xu G, Wu Q, Zhang Y (2018). Lysosomal deposition of copper oxide nanoparticles triggers HUVEC cells death. Biomaterials.

[98] Khan MI, Mohammad A, Patil G, Naqvi S, a H, Chauhan LKS, Ahmad I (2012). Induction of ROS, mitochondrial damage and autophagy in lung epithelial cancer cells by iron oxide nanoparticles. Biomaterials.

[99] Lopes VR, Loitto V, Audinot J-N, Bayat N, Gutleb AC, Cristobal S (2016). Dose-dependent autophagic effect of titanium dioxide nanoparticles in human HaCaT cells at non-cytotoxic levels. J Nanobiotechnol.

[100] Shi S, Lin S, Li Y, Zhang T, Shao X, Tian T (2018). Effects of tetrahedral DNA nanostructures on autophagy in chondrocytes. Chem Commun (Camb).

[101] Ivanovic V, Popovic D, Petrovic S, Rudolf R, Majerič P, Lazarevic M (2022). Unraveling the Antibiofilm activity of a New Nanogold Resin for Dentures and Epithesis. Pharmaceutics.

[102] Zhang Y, Wang P, Wang Y, Li J, Qiao D, Chen R (2021). Gold nanoparticles promote the bone regeneration of Periodontal Ligament Stem Cell Sheets through activation of Autophagy. Int J Nanomedicine.

[103] Croes M, Bakhshandeh S, van Hengel IaJ, Lietaert K, van Kessel KPM, Pouran B (2018). Antibacterial and immunogenic behavior of silver coatings on additively manufactured porous titanium. Acta Biomater.

[104] Chen Y, Guan M, Ren R, Gao C, Cheng H, Li Y (2020). Improved immunoregulation of Ultra-Low-Dose Silver nanoparticle-loaded TiO2 nanotubes via M2 macrophage polarization by regulating GLUT1 and autophagy. Int J Nanomedicine.

[105] Wu Y, Zhang C, Guo R, Wu D, Shi J, Li L (2021). Mesenchymal stem cells: an overview of their potential in cell-based therapy for Diabetic Nephropathy. Stem Cells Int.

[106] Yang B, Ding L, Yao H, Chen Y, Shi J (2020). A Metal-Organic Framework (MOF) Fenton Nanoagent-Enabled Nanocatalytic Cancer Therapy in Synergy with Autophagy Inhibition. Adv Mater.

[107] Zhu X, Ji X, Kong N, Chen Y, Mahmoudi M, Xu X (2018). Intracellular mechanistic understanding of 2D MoS2 nanosheets for Anti-Exocytosis-Enhanced Synergistic Cancer Therapy. ACS Nano.

[108] Zhang Z-H, Wu Q-Y, Zheng R, Chen C, Chen Y, Liu Q (2017). Selenomethionine mitigates Cognitive decline by targeting both tau hyperphosphorylation and autophagic clearance in an Alzheimer’s Disease Mouse Model. J Neurosci.

[109] Guo L, He N, Zhao Y, Liu T, Deng Y (2020). Autophagy modulated by Inorganic Nanomaterials. Theranostics.

[110] Demir E, Nedzvetsky VS, Ağca CA, Kirici M (2020). Pristine C60 fullerene nanoparticles ameliorate Hyperglycemia-Induced Disturbances via Modulation of apoptosis and autophagy flux. Neurochem Res.

[111] Zhang X, Wei C, Li Y, Li Y, Chen G, He Y (2020). Dose-dependent cytotoxicity induced by pristine graphene oxide nanosheets for potential bone tissue regeneration. J Biomed Mater Res A.

[112] Wei M, Fu Z, Wang C, Zheng W, Li S, Le W (2019). Graphene Oxide Nanocolloids Induce Autophagy-Lysosome dysfunction in mouse embryonic stem cells. J Biomed Nanotechnol.

[113] Mytych J, Wnuk M, Rattan SIS (2016). Low doses of nanodiamonds and silica nanoparticles have beneficial hormetic effects in normal human skin fibroblasts in culture. Chemosphere.

[114] Rahman MM, Uson-Lopez RA, Sikder MT, Tan G, Hosokawa T, Saito T (2018). Ameliorative effects of selenium on arsenic-induced cytotoxicity in PC12 cells via modulating autophagy/apoptosis. Chemosphere.

[115] Du S, Li C, Lu Y, Lei X, Zhang Y, Li S (2019). Dioscin alleviates crystalline Silica-Induced Pulmonary inflammation and fibrosis through promoting alveolar macrophage autophagy. Theranostics.

[116] Ha S-W, Weitzmann MN, Beck GR (2014). Bioactive silica nanoparticles promote osteoblast differentiation through stimulation of autophagy and direct association with LC3 and p62. ACS Nano.

[117] Chen P, Xia C, Mei S, Wang J, Shan Z, Lin X (2016). Intra-articular delivery of sinomenium encapsulated by chitosan microspheres and photo-crosslinked GelMA hydrogel ameliorates osteoarthritis by effectively regulating autophagy. Biomaterials.

[118] Li Y, Jiang S, Song L, Yao Z, Zhang J, Wang K (2021). Zwitterionic Hydrogel activates Autophagy to promote Extracellular Matrix Remodeling for Improved pressure Ulcer Healing. Front Bioeng Biotechnol.

[119] Yu Q, Hu X, Ma Y, Xie Y, Lu Y, Qi J (2016). Lipids-based nanostructured lipid carriers (NLCs) for improved oral bioavailability of sirolimus. Drug Deliv.

[120] Franck CO, Fanslau L, Bistrovic Popov A, Tyagi P, Fruk L (2021). Biopolymer-based carriers for DNA Vaccine Design. Angew Chem Int Ed Engl.

[121] Yang K, Lu Y, Xie F, Zou H, Fan X, Li B (2016). Cationic liposomes induce cell necrosis through lysosomal dysfunction and late-stage autophagic flux inhibition. Nanomed (Lond).

[122] Gao J, Ochyl LJ, Yang E, Moon JJ (2017). Cationic liposomes promote antigen cross-presentation in dendritic cells by alkalizing the lysosomal pH and limiting the degradation of antigens. Int J Nanomedicine.

[123] Lu N, Liu J, Tian Y, Liao M, Wang H, Lu Y (2014). Atg5 deficit exaggerates the lysosome formation and cathepsin B activation in mice brain after lipid nanoparticles injection. Nanomedicine.

[124] Chen X, Yu Q, Liu Y, Sheng Q, Shi K, Wang Y (2019). Synergistic cytotoxicity and co-autophagy inhibition in pancreatic tumor cells and cancer-associated fibroblasts by dual functional peptide-modified liposomes. Acta Biomater.

[125] Xie F, Zhang S, Liu J, Gong Z, Yang K, Zhang H (2016). Codelivery of salinomycin and chloroquine by liposomes enables synergistic antitumor activity in vitro. Nanomed (Lond).

[126] Zhang D, Atochina-Vasserman EN, Maurya DS, Huang N, Xiao Q, Ona N (2021). One-component multifunctional sequence-defined Ionizable Amphiphilic Janus Dendrimer Delivery Systems for mRNA. J Am Chem Soc.

[127] Zhang C, Shang Y, Chen X, Midgley AC, Wang Z, Zhu D (2020). Supramolecular nanofibers containing arginine-Glycine-aspartate (RGD) peptides boost therapeutic efficacy of Extracellular vesicles in kidney repair. ACS Nano.

[128] Wu L, Xie W, Li Y, Ni Q, Timashev P, Lyu M (2022). Biomimetic Nanocarriers Guide Extracellular ATP homeostasis to Remodel Energy Metabolism for activating Innate and adaptive immunity system. Adv Sci (Weinh).

[129] Jorge AF, Eritja R (2018). Overview of DNA Self-Assembling: progresses in Biomedical Applications. Pharmaceutics.

[130] Carter-Fenk K, Lao KU, Herbert JM (2021). Predicting and understanding non-covalent interactions using novel forms of symmetry-adapted perturbation theory. Acc Chem Res.

[131] Green LN, Subramanian HKK, Mardanlou V, Kim J, Hariadi RF, Franco E (2019). Autonomous dynamic control of DNA nanostructure self-assembly. Nat Chem.

[132] Zheng Y, Lei Y, Hu C, Hu C (2016). p53 regulates autophagic activity in senescent rat mesenchymal stromal cells. Exp Gerontol.

[133] Wen X, Wu J, Wang F, Liu B, Huang C, Wei Y (2013). Deconvoluting the role of reactive oxygen species and autophagy in human diseases. Free Radic Biol Med.

[134] Hulea L, Markovic Z, Topisirovic I, Simmet T, Trajkovic V (2016). Biomedical potential of mTOR modulation by nanoparticles. Trends Biotechnol.

[135] Popp L, Tran V, Patel R, Segatori L (2018). Autophagic response to cellular exposure to titanium dioxide nanoparticles. Acta Biomater.

[136] Du J, Xu Z, Liu Q, Yang Y, Qian H, Hu M (2017). ATG101 single-stranded antisense RNA-Loaded triangular DNA nanoparticles Control Human Pulmonary Endothelial Growth via Regulation of Cell Macroautophagy. ACS Appl Mater Interfaces.

[137] Wang H, Ma Y, Li J, Zhou C, Xu A, Xu Y (2022). Modulating autophagy by strontium-doped micro/nano rough titanium surface for promotion of osteogenesis and inhibition of osteoclastogenesis. Colloids Surf B Biointerfaces.

[138] Zhang X, Cui J, Cheng L, Lin K (2021). Enhancement of osteoporotic bone regeneration by strontium-substituted 45S5 bioglass via time-dependent modulation of autophagy and the Akt/mTOR signaling pathway. J Mater Chem B.

[139] Fan D, Liu H, Zhang Z, Su M, Yuan Z, Lin Y (2021). Resveratrol and Angiogenin-2 combined with PEGDA/TCS hydrogel for the targeted therapy of hypoxic bone defects via activation of the Autophagy Pathway. Front Pharmacol.

[140] Ruolan W, Liangjiao C, Longquan S (2020). The mTOR/ULK1 signaling pathway mediates the autophagy-promoting and osteogenic effects of dicalcium silicate nanoparticles. J Nanobiotechnol.

[141] Zhang Z, Fu X, Xu L, Hu X, Deng F, Yang Z (2020). Nanosized Alumina particle and proteasome inhibitor Bortezomib prevented inflammation and Osteolysis Induced by Titanium Particle via Autophagy and NF-κB signaling. Sci Rep.

[142] Chen M, Hu Y, Hou Y, Li M, Chen M, Mu C (2019). Differentiation regulation of mesenchymal stem cells via autophagy induced by structurally-different silica based nanobiomaterials. J Mater Chem B.

[143] Wang R, Hu H, Guo J, Wang Q, Cao J, Wang H (2019). Nano-Hydroxyapatite modulates osteoblast differentiation through autophagy induction via mTOR Signaling Pathway. J Biomed Nanotechnol.

[144] Wu J, Kuang L, Chen C, Yang J, Zeng W-N, Li T (2019). Mir-100-5p-abundant exosomes derived from infrapatellar fat pad MSCs protect articular cartilage and ameliorate gait abnormalities via inhibition of mTOR in osteoarthritis. Biomaterials.

[145] Jiao D, Wang J, Yu W, Zhang N, Zhang K, Bai Y (2021). Gelatin reduced Graphene Oxide Nanosheets as Kartogenin Nanocarrier induces rat ADSCs chondrogenic differentiation combining with Autophagy Modification. Mater (Basel).

[146] Shen S, Li L, Li S, Bai Y, Liu H (2018). Metal-organic frameworks induce autophagy in mouse embryonic fibroblast cells. Nanoscale.

[147] An Y, Liu WJ, Xue P, Ma Y, Zhang LQ, Zhu B (2018). Autophagy promotes MSC-mediated vascularization in cutaneous wound healing via regulation of VEGF secretion. Cell Death Dis.

[148] Qian Y, Han Q, Zhao X, Song J, Cheng Y, Fang Z (2018). 3D melatonin nerve scaffold reduces oxidative stress and inflammation and increases autophagy in peripheral nerve regeneration. J Pineal Res.

[149] Luo Q, Lin Y-X, Yang P-P, Wang Y, Qi G-B, Qiao Z-Y (2018). A self-destructive nanosweeper that captures and clears amyloid β-peptides. Nat Commun.

[150] Liu J, Liu C, Zhang J, Zhang Y, Liu K, Song J-X (2020). A self-assembled α-Synuclein nanoscavenger for Parkinson’s Disease. ACS Nano.

[151] Xiao L, Wei F, Zhou Y, Anderson GJ, Frazer DM, Lim YC (2020). Dihydrolipoic Acid-Gold Nanoclusters regulate Microglial polarization and have the potential to alter neurogenesis. Nano Lett.

[152] Wang L, Liu X, Fu J, Ning X, Zhang M, Jiang Z (2019). Release of methylene blue from graphene oxide-coated electrospun nanofibrous scaffolds to modulate functions of neural progenitor cells. Acta Biomater.

[153] Li D, Huang S, Zhu J, Hu T, Han Z, Zhang S (2019). Exosomes from MiR-21-5p-Increased neurons play a role in Neuroprotection by suppressing Rab11a-Mediated neuronal Autophagy in Vitro after traumatic brain Injury. Med Sci Monit.

[154] Wei P-F, Zhang L, Nethi SK, Barui AK, Lin J, Zhou W (2014). Accelerating the clearance of mutant huntingtin protein aggregates through autophagy induction by europium hydroxide nanorods. Biomaterials.

[155] Shen Y, Gong S, Li J, Wang Y, Zhang X, Zheng H (2019). Co-loading antioxidant N-acetylcysteine attenuates cytotoxicity of iron oxide nanoparticles in hypoxia/reoxygenation cardiomyocytes. Int J Nanomedicine.

[156] Bibee KP, Cheng Y-J, Ching JK, Marsh JN, Li AJ, Keeling RM (2014). Rapamycin nanoparticles target defective autophagy in muscular dystrophy to enhance both strength and cardiac function. FASEB J.

[157] Liu L, Xu Q, Zhang L, Sun H, Ding F, Li Y (2021). Fe3O4 magnetic nanoparticles ameliorate albumin-induced tubulointerstitial fibrosis by autophagy related to Rab7. Colloids Surf B Biointerfaces.

[158] An JH, Hong S-E, Yu S-L, Kang J, Park CG, Lee HY (2022). Ceria-Zirconia nanoparticles reduce intracellular globotriaosylceramide accumulation and attenuate kidney injury by enhancing the autophagy flux in cellular and animal models of fabry disease. J Nanobiotechnol.

[159] You Z, Qian H, Wang C, He B, Yan J, Mao C (2015). Regulation of vascular smooth muscle cell autophagy by DNA nanotube-conjugated mTOR siRNA. Biomaterials.

[160] Lee S, Han D, Kang H-G, Jeong SJ, Jo J-E, Shin J (2019). Intravenous sustained-release nifedipine ameliorates nonalcoholic fatty liver disease by restoring autophagic clearance. Biomaterials.

[161] Xu Y, Li Y, Liu X, Pan Y, Sun Z, Xue Y (2019). SPIONs enhances IL-10-producing macrophages to relieve sepsis via Cav1-Notch1/HES1-mediated autophagy. Int J Nanomedicine.

[162] Yin X, Zhou C, Li J, Liu R, Shi B, Yuan Q (2019). Autophagy in bone homeostasis and the onset of osteoporosis. Bone Res.

[163] Li Y, Liu C (2017). Nanomaterial-based bone regeneration. Nanoscale.

[164] Pang L, Jin H, Lu Z, Xie F, Shen H, Li X (2023). Treatment with mesenchymal stem cell-derived nanovesicle-containing gelatin methacryloyl hydrogels alleviates osteoarthritis by modulating chondrogenesis and macrophage polarization. Adv Healthc Mater.

[165] Mizushima N, Levine B (2010). Autophagy in mammalian development and differentiation. Nat Cell Biol.

[166] Rambold AS, Cohen S, Lippincott-Schwartz J (2015). Fatty acid trafficking in starved cells: regulation by lipid droplet lipolysis, autophagy, and mitochondrial fusion dynamics. Dev Cell.

[167] Shapiro IM, Layfield R, Lotz M, Settembre C, Whitehouse C (2014). Boning up on autophagy: the role of autophagy in skeletal biology. Autophagy.

[168] Ezzat S, Louka ML, Zakaria ZM, Nagaty MM, Metwaly RG (2019). Autophagy in osteoporosis: relation to oxidative stress. J Cell Biochem.

[169] Nuschke A, Rodrigues M, Stolz DB, Chu CT, Griffith L, Wells A (2014). Human mesenchymal stem cells/multipotent stromal cells consume accumulated autophagosomes early in differentiation. Stem Cell Res Ther.

[170] Wan Y, Zhuo N, Li Y, Zhao W, Jiang D (2017). Autophagy promotes osteogenic differentiation of human bone marrow mesenchymal stem cell derived from osteoporotic vertebrae. Biochem Biophys Res Commun.

[171] Girón J, Kerstner E, Medeiros T, Oliveira L, Machado GM, Malfatti CF (2021). Biomaterials for bone regeneration: an orthopedic and dentistry overview. Braz J Med Biol Res.

[172] Song W, Shi M, Dong M, Zhang Y (2016). Inducing temporal and reversible autophagy by Nanotopography for potential control of cell differentiation. ACS Appl Mater Interfaces.

[173] Xian G, Chen W, Gu M, Ye Y, Yang G, Lai W (2020). Titanium particles induce apoptosis by promoting autophagy in macrophages via the PI3K/Akt signaling pathway. J Biomed Mater Res A.

[174] Wei F, Li M, Crawford R, Zhou Y, Xiao Y (2019). Exosome-integrated titanium oxide nanotubes for targeted bone regeneration. Acta Biomater.

[175] Kim S, Lee M (2020). Rational design of hydrogels to enhance osteogenic potential. Chem Mater.

[176] Xiao L, Shiwaku Y, Hamai R, Tsuchiya K, Sasaki K, Suzuki O (2021). Macrophage polarization related to crystal phases of calcium phosphate biomaterials. Int J Mol Sci.

[177] Li X, Xu J, Dai B, Wang X, Guo Q, Qin L (2020). Targeting autophagy in osteoporosis: from pathophysiology to potential therapy. Ageing Res Rev.

[178] Wang Y, Wang Y, Wu J, Liu C, Zhou Y, Mi L (2019). PRAK is required for the formation of Neutrophil Extracellular Traps. Front Immunol.

[179] Takayama K, Kawakami Y, Kobayashi M, Greco N, Cummins JH, Matsushita T (2014). Local intra-articular injection of rapamycin delays articular cartilage degeneration in a murine model of osteoarthritis. Arthritis Res Ther.

[180] Pape E, Parent M, Pinzano A, Sapin-Minet A, Henrionnet C, Gillet P (2021). Rapamycin-loaded poly(lactic-co-glycolic) acid nanoparticles: Preparation, characterization, and in vitro toxicity study for potential intra-articular injection. Int J Pharm.

[181] Ou L, Song B, Liang H, Liu J, Feng X, Deng B (2016). Toxicity of graphene-family nanoparticles: a general review of the origins and mechanisms. Part Fibre Toxicol.

[182] Raucci MG, Giugliano D, Longo A, Zeppetelli S, Carotenuto G, Ambrosio L (2017). Comparative facile methods for preparing graphene oxide-hydroxyapatite for bone tissue engineering. J Tissue Eng Regen Med.

[183] Bortz DR, Heras EG, Martin-Gullon I (2012). Impressive fatigue life and fracture toughness improvements in Graphene Oxide/Epoxy Composites. Macromolecules.

[184] Moradi L, Vasei M, Dehghan MM, Majidi M, Farzad Mohajeri S, Bonakdar S (2017). Regeneration of meniscus tissue using adipose mesenchymal stem cells-chondrocytes co-culture on a hybrid scaffold: in vivo study. Biomaterials.

[185] Toh WS, Lai RC, Hui JHP, Lim SK (2017). MSC exosome as a cell-free MSC therapy for cartilage regeneration: implications for osteoarthritis treatment. Semin Cell Dev Biol.

[186] Xia C, Chen P, Mei S, Ning L, Lei C, Wang J (2017). Photo-crosslinked HAMA hydrogel with cordycepin encapsulated chitosan microspheres for osteoarthritis treatment. Oncotarget.

[187] Li W, Bei Y, Pan X, Zhu J, Zhang Z, Zhang T (2023). Selenide-linked polydopamine-reinforced hybrid hydrogels with on-demand degradation and light-triggered nanozyme release for diabetic wound healing. Biomater Res.

[188] Wu Y, Su M, Zhang S, Xiao L, Xiao Y, Zhang M (2023). A mesenchymal stem cell-derived nanovesicle-biopotentiated bovine serum albumin-bridged gelatin hydrogel for enhanced diabetic wound therapy. Mater Design.

[189] Kawano A, Ariyoshi W, Yoshioka Y, Hikiji H, Nishihara T, Okinaga T (2019). Docosahexaenoic acid enhances M2 macrophage polarization via the p38 signaling pathway and autophagy. J Cell Biochem.

[190] Qiang L, Yang S, Cui Y-H, He Y-Y (2021). Keratinocyte autophagy enables the activation of keratinocytes and fibroblastsand facilitates wound healing. Autophagy.

[191] Zhou L, Liu Z, Chen S, Qiu J, Li Q, Wang S (2021). Transcription factor EB–mediated autophagy promotes dermal fibroblast differentiation and collagen production by regulating endoplasmic reticulum stress and autophagy–dependent secretion. Int J Mol Med.

[192] Zarei F, Soleimaninejad M (2018). Role of growth factors and biomaterials in wound healing. Artif Cells Nanomed Biotechnol.

[193] Feng X, Zhang Y, Zhang C, Lai X, Zhang Y, Wu J (2020). Nanomaterial-mediated autophagy: coexisting hazard and health benefits in biomedicine. Part Fibre Toxicol.

[194] Bainbridge P (2013). Wound healing and the role of fibroblasts. J Wound Care.

[195] Ali T, Rahman SU, Hao Q, Li W, Liu Z, Ali Shah F (2020). Melatonin prevents neuroinflammation and relieves depression by attenuating autophagy impairment through FOXO3a regulation. J Pineal Res.

[196] Li L, Chen X, Gu H (2016). The signaling involved in autophagy machinery in keratinocytes and therapeutic approaches for skin diseases. Oncotarget.

[197] Moreira HR, Marques AP (2022). Vascularization in skin wound healing: where do we stand and where do we go?. Curr Opin Biotechnol.

[198] DiPietro LA (2016). Angiogenesis and wound repair: when enough is enough. J Leukoc Biol.

[199] Mameli E, Martello A, Caporali A (2022). Autophagy at the interface of endothelial cell homeostasis and vascular disease. FEBS J.

[200] Han J, Pan X-Y, Xu Y, Xiao Y, An Y, Tie L (2012). Curcumin induces autophagy to protect vascular endothelial cell survival from oxidative stress damage. Autophagy.

[201] Li D, Ding Z, Du K, Ye X, Cheng S (2021). Reactive oxygen species as a link between antioxidant pathways and autophagy. Oxid Med Cell Longev.

[202] Wang Y, Wu Y, Wang Y, Xu H, Mei X, Yu D (2017). Antioxidant Properties of probiotic Bacteria. Nutrients.

[203] Deng L, Du C, Song P, Chen T, Rui S, Armstrong DG (2021). The role of oxidative stress and Antioxidants in Diabetic Wound Healing. Oxid Med Cell Longev.

[204] Zhang S, Li Y, Qiu X, Jiao A, Luo W, Lin X (2021). Incorporating redox-sensitive nanogels into bioabsorbable nanofibrous membrane to acquire ROS-balance capacity for skin regeneration. Bioactive Mater.

[205] Yamamoto A, Yue Z (2014). Autophagy and its normal and pathogenic states in the brain. Annu Rev Neurosci.

[206] Menzies FM, Fleming A, Caricasole A, Bento CF, Andrews SP, Ashkenazi A (2017). Autophagy and neurodegeneration: pathogenic Mechanisms and Therapeutic Opportunities. Neuron.

[207] Wu J, Lipinski MM (2019). Autophagy in Neurotrauma: good, bad, or Dysregulated. Cells.

[208] Fleming A, Rubinsztein DC (2020). Autophagy in neuronal development and plasticity. Trends Neurosci.

[209] Jang SY, Shin YK, Park SY, Park JY, Lee HJ, Yoo YH (2016). Autophagic myelin destruction by Schwann cells during wallerian degeneration and segmental demyelination. Glia.

[210] Jarrin S, Cabré S, Dowd E (2021). The potential of biomaterials for central nervous system cellular repair. Neurochem Int.

[211] Amani H, Kazerooni H, Hassanpoor H, Akbarzadeh A, Pazoki-Toroudi H (2019). Tailoring synthetic polymeric biomaterials towards nerve tissue engineering: a review. Artif Cells Nanomed Biotechnol.

[212] Qian Y, Lin H, Yan Z, Shi J, Fan C (2021). Functional nanomaterials in peripheral nerve regeneration: Scaffold design, chemical principles and microenvironmental remodeling. Mater Today.

[213] Gao J, Chen X, Ma T, He B, Li P, Zhao Y (2020). PEG-Ceramide nanomicelles induce autophagy and degrade tau proteins in N2a cells. Int J Nanomedicine.

[214] Knight AL, Yan X, Hamamichi S, Ajjuri RR, Mazzulli JR, Zhang MW (2014). The glycolytic enzyme, GPI, is a functionally conserved modifier of dopaminergic neurodegeneration in Parkinson’s models. Cell Metab.

[215] Settembre C, Fraldi A, Medina DL, Ballabio A (2013). Signals from the lysosome: a control centre for cellular clearance and energy metabolism. Nat Rev Mol Cell Biol.

[216] Bourdenx M, Daniel J, Genin E, Soria FN, Blanchard-Desce M, Bezard E (2016). Nanoparticles restore lysosomal acidification defects: implications for Parkinson and other lysosomal-related diseases. Autophagy.

[217] Geuna S, Raimondo S, Ronchi G, Di Scipio F, Tos P, Czaja K (2009). Chapter 3: histology of the peripheral nerve and changes occurring during nerve regeneration. Int Rev Neurobiol.

[218] Burnett MG, Zager EL (2004). Pathophysiology of peripheral nerve injury: a brief review. Neurosurg Focus.

[219] Faroni A, Mobasseri SA, Kingham PJ, Reid AJ (2015). Peripheral nerve regeneration: experimental strategies and future perspectives. Adv Drug Deliv Rev.

[220] Jiang M, Wang H, Jin M, Yang X, Ji H, Jiang Y (2018). Exosomes from MiR-30d-5p-ADSCs reverse Acute Ischemic Stroke-Induced, autophagy-mediated Brain Injury by promoting M2 Microglial/Macrophage polarization. Cell Physiol Biochem.

[221] Li Y, Zhu H, Wang S, Qian X, Fan J, Wang Z (2015). Interplay of oxidative stress and autophagy in PAMAM Dendrimers-Induced neuronal cell death. Theranostics.

[222] Isomi M, Sadahiro T, Ieda M (2019). Progress and Challenge of Cardiac Regeneration to treat heart failure. J Cardiol.

[223] Sciarretta S, Maejima Y, Zablocki D, Sadoshima J (2018). The role of Autophagy in the heart. Annu Rev Physiol.

[224] Kuma A, Hatano M, Matsui M, Yamamoto A, Nakaya H, Yoshimori T (2004). The role of autophagy during the early neonatal starvation period. Nature.

[225] Kanamori H, Takemura G, Goto K, Maruyama R, Ono K, Nagao K (2011). Autophagy limits acute myocardial infarction induced by permanent coronary artery occlusion. Am J Physiol Heart Circ Physiol.

[226] Sun Y, Yao X, Zhang Q-J, Zhu M, Liu Z-P, Ci B (2018). Beclin-1-Dependent Autophagy protects the Heart during Sepsis. Circulation.

[227] Wu X, Liu Z, Yu X-Y, Xu S, Luo J (2021). Autophagy and cardiac diseases: therapeutic potential of natural products. Med Res Rev.

[228] Yamamoto T, Takabatake Y, Kimura T, Takahashi A, Namba T, Matsuda J (2016). Time-dependent dysregulation of autophagy: implications in aging and mitochondrial homeostasis in the kidney proximal tubule. Autophagy.

[229] Hartleben B, Gödel M, Meyer-Schwesinger C, Liu S, Ulrich T, Köbler S (2010). Autophagy influences glomerular disease susceptibility and maintains podocyte homeostasis in aging mice. J Clin Invest.

[230] Lin T-A, Wu VC-C, Wang C-Y (2019). Autophagy in Chronic Kidney Diseases Cells.

[231] Feere DA, Velenosi TJ, Urquhart BL (2015). Effect of erythropoietin on hepatic cytochrome P450 expression and function in an adenine-fed rat model of chronic kidney disease. Br J Pharmacol.

[232] Klinkhammer BM, Goldschmeding R, Floege J, Boor P (2017). Treatment of renal fibrosis-turning Challenges into Opportunities. Adv Chronic Kidney Dis.

[233] Breyer MD, Susztak K (2016). The next generation of therapeutics for chronic kidney disease. Nat Rev Drug Discov.

[234] Zeisberg M, Zeisberg EM (2015). Precision renal medicine: a roadmap towards targeted kidney fibrosis therapies. Fibrogenesis Tissue Repair.

[235] Kimura T, Takabatake Y, Takahashi A, Kaimori J, Matsui I, Namba T (2011). Autophagy protects the proximal tubule from degeneration and acute ischemic injury. J Am Soc Nephrol.

[236] Takahashi A, Kimura T, Takabatake Y, Namba T, Kaimori J, Kitamura H (2012). Autophagy guards against cisplatin-induced acute kidney injury. Am J Pathol.

[237] Lenoir O, Tharaux P-L, Huber TB (2016). Autophagy in kidney disease and aging: lessons from rodent models. Kidney Int.

[238] Sirolli V, Pieroni L, Di Liberato L, Urbani A, Bonomini M (2019). Urinary peptidomic biomarkers in kidney Diseases. Int J Mol Sci.

[239] Eardley KS, Zehnder D, Quinkler M, Lepenies J, Bates RL, Savage CO (2006). The relationship between albuminuria, MCP-1/CCL2, and interstitial macrophages in chronic kidney disease. Kidney Int.

[240] Hutton HL, Ooi JD, Holdsworth SR, Kitching AR (2016). The NLRP3 inflammasome in kidney disease and autoimmunity. Nephrol (Carlton).

[241] Lorenz G, Darisipudi MN, Anders H-J (2014). Canonical and non-canonical effects of the NLRP3 inflammasome in kidney inflammation and fibrosis. Nephrol Dial Transplant.

[242] Trümper V, Wittig I, Heidler J, Richter F, Brüne B, von Knethen A (2020). Redox Regulation of PPARγ in polarized macrophages. PPAR Res.

[243] Li Y-F, Ouyang S-H, Tu L-F, Wang X, Yuan W-L, Wang G-E (2018). Caffeine protects skin from oxidative Stress-Induced Senescence through the activation of Autophagy. Theranostics.

[244] Stevens T, Phan S, Frid MG, Alvarez D, Herzog E, Stenmark KR (2008). Lung vascular cell heterogeneity: endothelium, smooth muscle, and fibroblasts. Proc Am Thorac Soc.

[245] Sauler M, Bazan IS, Lee PJ (2019). Cell death in the lung: the apoptosis-necroptosis Axis. Annu Rev Physiol.

[246] X L, F Z, A W, P C, H C. Role and mechanisms of autophagy in lung metabolism and repair. Cellular and molecular life sciences: CMLS [Internet]. 2021 [cited 2023 May 14];78. Available from: https://pubmed.ncbi.nlm.nih.gov/33864479/. 10.1007/s00018-021-03841-7 PMC11072280 33864479

[247] Racanelli AC, Choi AMK, Choi ME (2020). Autophagy in chronic lung disease. Prog Mol Biol Transl Sci.

[248] FitzGerald ES, Luz NF, Jamieson AM (2020). Competitive cell death interactions in pulmonary infection: host modulation Versus Pathogen Manipulation. Front Immunol.

[249] Lee J-W, Chun W, Lee HJ, Min J-H, Kim S-M, Seo J-Y (2021). The role of Macrophages in the development of Acute and Chronic Inflammatory Lung Diseases. Cells.

[250] Chen S, Yang J, Wei Y, Wei X (2020). Epigenetic regulation of macrophages: from homeostasis maintenance to host defense. Cell Mol Immunol.

[251] Lu H-L, Huang X-Y, Luo Y-F, Tan W-P, Chen P-F, Guo Y-B (2018). Activation of M1 macrophages plays a critical role in the initiation of acute lung injury. Biosci Rep.

[252] Goncharov DA, Kudryashova TV, Ziai H, Ihida-Stansbury K, DeLisser H, Krymskaya VP (2014). Mammalian target of rapamycin complex 2 (mTORC2) coordinates pulmonary artery smooth muscle cell metabolism, proliferation, and survival in pulmonary arterial hypertension. Circulation.

[253] Mizumura K, Cloonan SM, Haspel JA, Choi AMK (2012). The emerging importance of autophagy in pulmonary diseases. Chest.

[254] Guignabert C, Tu L, Girerd B, Ricard N, Huertas A, Montani D (2015). New molecular targets of pulmonary vascular remodeling in pulmonary arterial hypertension: importance of endothelial communication. Chest.

[255] Aggarwal S, Gross CM, Sharma S, Fineman JR, Black SM (2013). Reactive oxygen species in pulmonary vascular remodeling. Compr Physiol.

[256] Qian H, Chao X, Williams J, Fulte S, Li T, Yang L (2021). Autophagy in liver diseases: a review. Mol Aspects Med.

[257] Ke P-Y (2019). Diverse functions of Autophagy in Liver Physiology and Liver Diseases. IJMS.

